# Neurological outcomes in immune checkpoint inhibitor-related neurotoxicity

**DOI:** 10.1093/braincomms/fcad169

**Published:** 2023-05-27

**Authors:** Antonio Farina, Cristina Birzu, Mad-Hélénie Elsensohn, Alberto Picca, Sergio Muñiz-Castrillo, Alberto Vogrig, Macarena Villagrán-García, Nicolás Lundahl Ciano-Petersen, Luca Massacesi, Baptiste Hervier, Sarah Guégan, Nora Kramkimel, Yann Vano, Joe Elie Salem, Yves Allenbach, Thierry Maisonobe, Souad Assaad, Aurélien Maureille, Perrine Devic, Nicolas Weiss, Antoine Pegat, Delphine Maucort-Boulch, Damien Ricard, Jérôme Honnorat, Dimitri Psimaras, Bastien Joubert

**Affiliations:** Reference Centre for Paraneoplastic Neurological Syndromes and Autoimmune Encephalitis, Hospices Civils de Lyon, Neurological Hospital, Bron 69677, France; MeLiS - UCBL-CNRS UMR 5284—INSERM U1314, Université Claude Bernard Lyon 1, Lyon 69008, France; Department of Neuroscience, Psychology, Pharmacology and Child Health, University of Florence, Florence 50139, Italy; Department of Neurology 2 Mazarin, Sorbonne University, Brain Institute, INSERM UMR 1127, Groupe Hospitalier Pitié-Salpêtrière, Paris 75013, France; OncoNeuroTox Group, Center for Patients with Neurological Complications of Oncologic Treatments Groupe Hospitalier Pitié-Salpêtrière et Hôpital Percy, Paris 75561, France; Biostatistics—Bioinformatics Department, Public Health Unit. Hospices Civils de Lyon, Lyon 69003, France; Laboratory of Biometry and Evolutionary Biology, University Claude Bernard Lyon 1, Villeurbanne 69622, France; CNRS, UMR5558, Laboratory of Biometry and Evolutionary Biology, Biostatistics-Health Team, Villeurbanne 69622, France; Department of Neurology 2 Mazarin, Sorbonne University, Brain Institute, INSERM UMR 1127, Groupe Hospitalier Pitié-Salpêtrière, Paris 75013, France; OncoNeuroTox Group, Center for Patients with Neurological Complications of Oncologic Treatments Groupe Hospitalier Pitié-Salpêtrière et Hôpital Percy, Paris 75561, France; Reference Centre for Paraneoplastic Neurological Syndromes and Autoimmune Encephalitis, Hospices Civils de Lyon, Neurological Hospital, Bron 69677, France; MeLiS - UCBL-CNRS UMR 5284—INSERM U1314, Université Claude Bernard Lyon 1, Lyon 69008, France; Reference Centre for Paraneoplastic Neurological Syndromes and Autoimmune Encephalitis, Hospices Civils de Lyon, Neurological Hospital, Bron 69677, France; MeLiS - UCBL-CNRS UMR 5284—INSERM U1314, Université Claude Bernard Lyon 1, Lyon 69008, France; Reference Centre for Paraneoplastic Neurological Syndromes and Autoimmune Encephalitis, Hospices Civils de Lyon, Neurological Hospital, Bron 69677, France; MeLiS - UCBL-CNRS UMR 5284—INSERM U1314, Université Claude Bernard Lyon 1, Lyon 69008, France; Reference Centre for Paraneoplastic Neurological Syndromes and Autoimmune Encephalitis, Hospices Civils de Lyon, Neurological Hospital, Bron 69677, France; MeLiS - UCBL-CNRS UMR 5284—INSERM U1314, Université Claude Bernard Lyon 1, Lyon 69008, France; Department of Neuroscience, Psychology, Pharmacology and Child Health, University of Florence, Florence 50139, Italy; Department of Internal Medicine, AP-HP, Hôpital St Louis, Paris 75010, France; Department of Dermatology, AP-HP, Hôpital Cochin, Paris 75014, France; Department of Dermatology, Université de Paris Cité, Paris 75006, France; Department of Dermatology, AP-HP, Hôpital Cochin, Paris 75014, France; Department of Medical Oncology, AP-HP, Centre Hôpital Européen Georges-Pompidou, Paris 75015, France; Department of Pharmacology, Sorbonne University, INSERM, UNICO-GRECO Cardio-oncology Program, CIC-1901, AP-HP, Hôpital Pitié-Salpêtrière, F-75013 Paris, France; APHP, Department of Internal Medicine, Sorbonne University, Sorbonne University, INSERM Groupe Hospitalier Pitié-Salpêtrière, Paris 75651, France; APHP, Department of Clinical Neurophysiology, Sorbonne University, Paris 75013, France; Department of Medical Oncology, Léon Bérard Cancer Center, Lyon 69008, France; Department of Medical Oncology, Léon Bérard Cancer Center, Lyon 69008, France; ImmuCare, Institute of Cancerology, Hospices Civils de Lyon, 69002 Lyon, France; Department of Neurology, Hôpital Lyon Sud, Hospices Civils de Lyon, Lyon 69495, France; Department of Neurology, Sorbonne University, Hôpital de la Pitié-Salpêtrière, unité de Médecine Intensive Réanimation à orientation neurologique, Paris 75013, France; Brain Liver Pitié-Salpêtrière (BLIPS) Study Group, INSERM UMR_S 938, Centre de recherche Saint-Antoine, Metabolic, Biliary and Fibro-Inflammatory Diseases of the Liver, Institute of Cardiometabolism and Nutrition (ICAN), Paris 75012, France; Department of Neurological Functional Explorations, Hospices Civils de Lyon, Neurological Hospital, Bron 69500, France; Biostatistics—Bioinformatics Department, Public Health Unit. Hospices Civils de Lyon, Lyon 69003, France; Laboratory of Biometry and Evolutionary Biology, University Claude Bernard Lyon 1, Villeurbanne 69622, France; CNRS, UMR5558, Laboratory of Biometry and Evolutionary Biology, Biostatistics-Health Team, Villeurbanne 69622, France; OncoNeuroTox Group, Center for Patients with Neurological Complications of Oncologic Treatments Groupe Hospitalier Pitié-Salpêtrière et Hôpital Percy, Paris 75561, France; Neurology Department, Hôpital d’Instruction des Armées Percy, Service de Santé des Armées, Clamart 92140, France; Reference Centre for Paraneoplastic Neurological Syndromes and Autoimmune Encephalitis, Hospices Civils de Lyon, Neurological Hospital, Bron 69677, France; MeLiS - UCBL-CNRS UMR 5284—INSERM U1314, Université Claude Bernard Lyon 1, Lyon 69008, France; ImmuCare, Institute of Cancerology, Hospices Civils de Lyon, 69002 Lyon, France; Department of Neurology 2 Mazarin, Sorbonne University, Brain Institute, INSERM UMR 1127, Groupe Hospitalier Pitié-Salpêtrière, Paris 75013, France; OncoNeuroTox Group, Center for Patients with Neurological Complications of Oncologic Treatments Groupe Hospitalier Pitié-Salpêtrière et Hôpital Percy, Paris 75561, France; Reference Centre for Paraneoplastic Neurological Syndromes and Autoimmune Encephalitis, Hospices Civils de Lyon, Neurological Hospital, Bron 69677, France; MeLiS - UCBL-CNRS UMR 5284—INSERM U1314, Université Claude Bernard Lyon 1, Lyon 69008, France; ImmuCare, Institute of Cancerology, Hospices Civils de Lyon, 69002 Lyon, France; Department of Neurology, Hôpital Lyon Sud, Hospices Civils de Lyon, Lyon 69495, France

**Keywords:** immune checkpoint inhibitors, cancer immunotherapy complications, neurotoxicity, neurological immune-related adverse events, paraneoplastic neurological syndromes

## Abstract

While the spectrum of neurological immune checkpoint inhibitor-related adverse events is expanding, patients’ outcomes are not well documented. This study aimed to assess outcomes of neurological immune-related adverse events and to identify prognostic factors. All patients experiencing grade ≥2 neurological immune-related adverse events identified at two clinical networks (French Reference Center for Paraneoplastic Neurological Syndromes, Lyon; and OncoNeuroTox, Paris) over five years were included. Modified Rankin scores were assessed at onset, 6, 12, 18 months, and last visit. A multi-state Markov model was used to estimate the transition rates between minor disability (mRS <3), severe disability (mRS 3-5), and death (mRS 6), over the study period. The state-to-state transition rates were estimated using maximum likelihood and variables were introduced into the different transitions to study their effects. A total of 147 patients were included out of 205 patients with a suspicion of neurological immune-related adverse events. The median age was 65 years (range 20–87) and 87/147 patients (59.2%) were male. Neurological immune-related adverse events involved the peripheral nervous system in 87/147 patients (59.2%), the central nervous system in 51/147 (34.7%), and both systems in 9/147 (6.1%). Paraneoplastic-like syndromes were observed in 30/147 patients (20.4%). Cancers included lung cancers (36.1%), melanoma (30.6%), urological cancers (15.6%), and others (17.8%). Patients were treated with programmed cell death protein (ligan) 1 (PD(L)1) inhibitors (70.1%), CTLA4 inhibitors (3.4%) or both (25.9%). Severe disability was reported in 108/144 patients (75.0%) at onset and in 33/146 patients (22.6%) at last visit (median follow-up duration: 12 months, range 0.5–50); 48/147 (32.7%) patients died, from cancer progression (17/48, 35.4%), neurological toxicity (15/48, 31.2%), other causes (10/48, 20.8%) or unknown causes (6/48, 12.5%). The rate of transition from severe to minor disability independently increased with melanoma [compared to lung cancer, hazard ratio = 3.26, 95%CI (1.27; 8.41)] and myositis/neuromuscular junction disorders [hazard ratio = 8.26, 95%CI (2.90; 23.58)], and decreased with older age [hazard ratio = 0.68, 95%CI (0.47; 0.99)] and paraneoplastic-like syndromes [hazard ratio = 0.29, 95%CI (0.09; 0.98)]. In patients with neurological immune-related adverse events, myositis/neuromuscular junction disorders and melanoma increase the transition rate from severe to minor disability, while older age and paraneoplastic-like syndromes result in poorer neurological outcomes; future studies are needed to optimize the management of such patients.

## Teaser

Farina *et al*. report 147 cases of neurological immune-related adverse events and find that neurological recovery is possible even in severe presentations, with myositis/neuromuscular junction disorders and melanoma associated with an increased rate of neurological recovery, while older age and paraneoplastic-like syndromes predict poor neurological outcome.

## Graphical Abstract

**Graphical Abstract:**
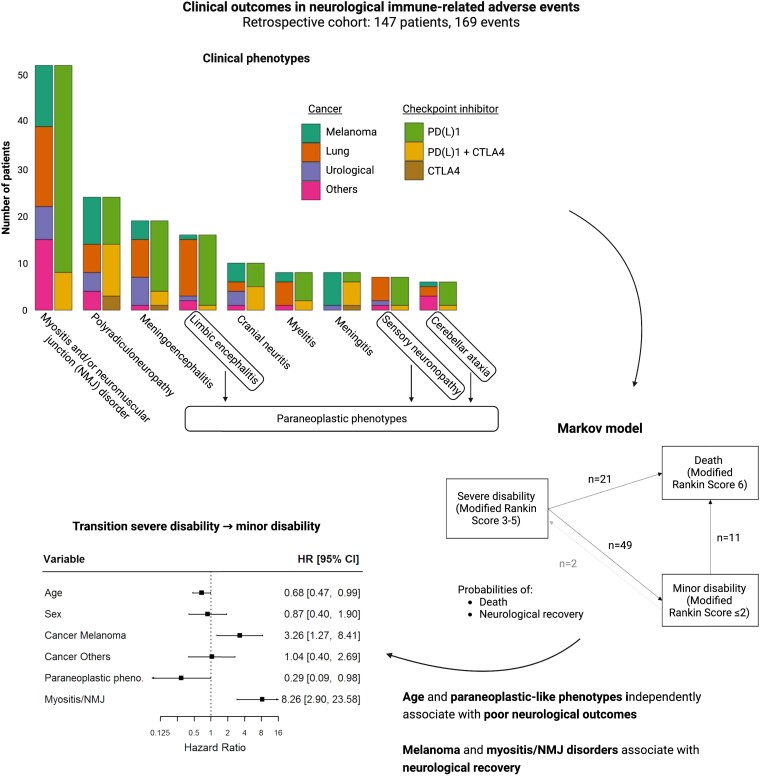

See Zeiser and Prinz (https://doi.org/10.1093/braincomms/fcad186) for a scientific commentary on this article.

## Introduction

The paradigms of cancer management have been transformed over the last decade with the advent of immune checkpoint inhibitors (ICIs).^1,2^ In contrast to traditional chemotherapy, these drugs target immune checkpoints such as cytotoxic T-lymphocyte antigen-4, programmed death-1 and/or programmed death ligand-1, thereby enhancing the ability of the host immune system to destroy tumour cells. ICIs have dramatically improved the survival of patients harbouring certain malignancies and their use is expanding in oncological clinical practice.^1–3^ In addition to the intended effect on anti-cancer immunity, ICIs may also break self-tolerance and induce novel immune-mediated toxicities known as immune-related adverse events (irAEs),^1^ including neurological irAEs (n-irAEs) that are reported in 1–5% of patients.^4–8^ The clinical spectrum of n-irAEs is broad, including myositis, myasthenic syndromes, peripheral neuropathies, and encephalitis, and can also mimic paraneoplastic neurological syndromes (PNS).^9^ N-irAEs are often severe and may be life-threatening,^8,10^ requiring permanent ICI discontinuation and intensive immunosuppression,^1,11^ and so far the impact on the risk of cancer progression remains unknown. Predicting the outcomes of n-irAE patients is challenging due to the heterogeneity of clinical presentations, limited availability of follow-up data, and the complex intersection between the neurological condition and underlying cancer. Herein, we studied a large multicenter retrospective cohort of patients in order to assess the outcome of n-irAEs and to identify prognostic factors.

## Materials and methods

### Study design, setting, and participants

The databases of two clinical networks in France (French Reference Center for PNS, Lyon, and OncoNeuroTox, Paris) were retrospectively screened to identify all ≥18 years old suspected cases of n-irAE between 1 January 2015 and 30 June 2021. N-irAE was suspected by the local treating physician, who ruled out alternative diagnoses. All available medical charts were centrally reviewed by at least two authors. All patients with a final diagnosis of common terminology criteria for adverse events (CTCAE) grade ≥2 n-irAEs were included. The diagnosis of n-irAEs was based on the temporal association with ICI administration (<6 months from the last ICI dose)^12^ and the comprehensive exclusion of alternative diagnoses, including cancer dissemination, toxicities of other oncological treatments, infectious and metabolic causes. Clinical data were obtained retrospectively from the available electronical medical charts of the patients. A set of clinical variables (demographics, oncological characteristics, neurological symptoms, paraclinical findings, and outcome measures) were identified a priori. The chart review was performed by one author (A.F.). Data accuracy was verified by a second co-author (C.B. or B.J.). In case of discrepant or missing data, information was requested by email to the referring physician. Available electroneuromyography (ENMG) recordings were re-analysed by one author (A.P.) blinded to clinical data. Neurological phenotypes were classified on the basis of clinical syndromes supported by consistent radiological or ENMG findings. Clinical presentations fulfilling the definition of high-risk phenotypes for PNS from the updated 2021 criteria ([.e. limbic encephalitis, sensory neuronopathy (SNN), rapidly progressive cerebellar ataxia, Lambert–Eaton myasthenic syndrome (LEMS), and others] were referred to as paraneoplastic-like syndromes.^13^ The definition of limbic encephalitis was based on previously published criteria.^14^ Anti-neural antibodies defined as high-risk for PNS in the same publication (i.e. Hu, CV2/CRMP5, SOX1, PCA2, amphiphysin, Ri, Yo, Ma2 and Tr/DNER, KLHL-11)^13^ were referred as paraneoplastic-related autoantibodies. The presence of anti-neural/glial antibodies was assessed by immunohistofluorescence on rat brain sections and a confirmatory test represented by line-blot analysis on recombinant proteins (Euroimmun, Lubeck, Germany and/or Ravo Diagnostika, Freiburg, Germany) and/or cell-based assays (in-house techniques), as reported elsewhere.^15^ Symptom progression was classified as acute (maximum severity reached in less than 24 hours), subacute (maximum severity reached in less than 3 months and more than 24 hours), or chronic (maximum severity reached in more than 3 months). Modified Rankin scale (mRS) scores were retrospectively assessed^16^ at onset, 6, 12, 18 months and at the last visit. CTCAE v5.0^17^ were also retrospectively assessed at the onset and last visit. Patients who died for causes other than n-irAEs or unknown causes were not included in the analysis of CTCAE grade at the last visit. N-irAE relapse was defined as the recurrence of the neurological symptoms, after sustained improvement lasting at least 4 weeks. Data were abstracted using a standardized electronic form (Filemaker database management software, Claris International, Sunnycale, CA, USA).

### Statistical analysis

Continuous data were expressed as median (range) and categorical data as count (percentage). Categorical data were compared using the Fisher exact test. In order to study the competitive risk of death and neurological recovery over the study period, a multi-state Markov model^18,19^ was used to assess the rate of transition between minor disability (mRS < 3), severe disability (mRS 3-5), and death (mRS = 6). State-to-state transition rates were estimated using maximum likelihood and clinically relevant variables were introduced into the different transitions to study their effects, including age, sex, cancer type (grouped into three categories: lung, melanoma or other), type of ICI, n-irAEs phenotype, paraneoplastic-related antibodies and associated non-neurological irAEs. Variables were included in the multivariate analysis depending on the findings of the univariate analysis and the presence of missing data (variables with >30% missing values were excluded from the model), to obtain the most parsimonious model resulting in numerical stability and generalizability of the results. In addition to the 95% confident intervals, *P*-values were calculated using the Wald test. Statistical analyses were performed using R, version 3.4.0 (R Foundation for Statistical Computing, Vienna, Austria). All *P*-values were two-tailed, and *P*-values <0.05 were considered statistically significant.

### Ethical considerations

Approval for this study was granted by the institutional review board of the *Université Claude Bernard Lyon 1* and *Hospices Civils de Lyon* (69HCL21-474), and the study was registered to the *Commission nationale de l’informatique et des libertés* (CNIL, 21-5474). Patients’ informed consent was obtained according to the Declaration of Helsinki and its later amendments.

## Results

### Cohort description

**Figure 1:**
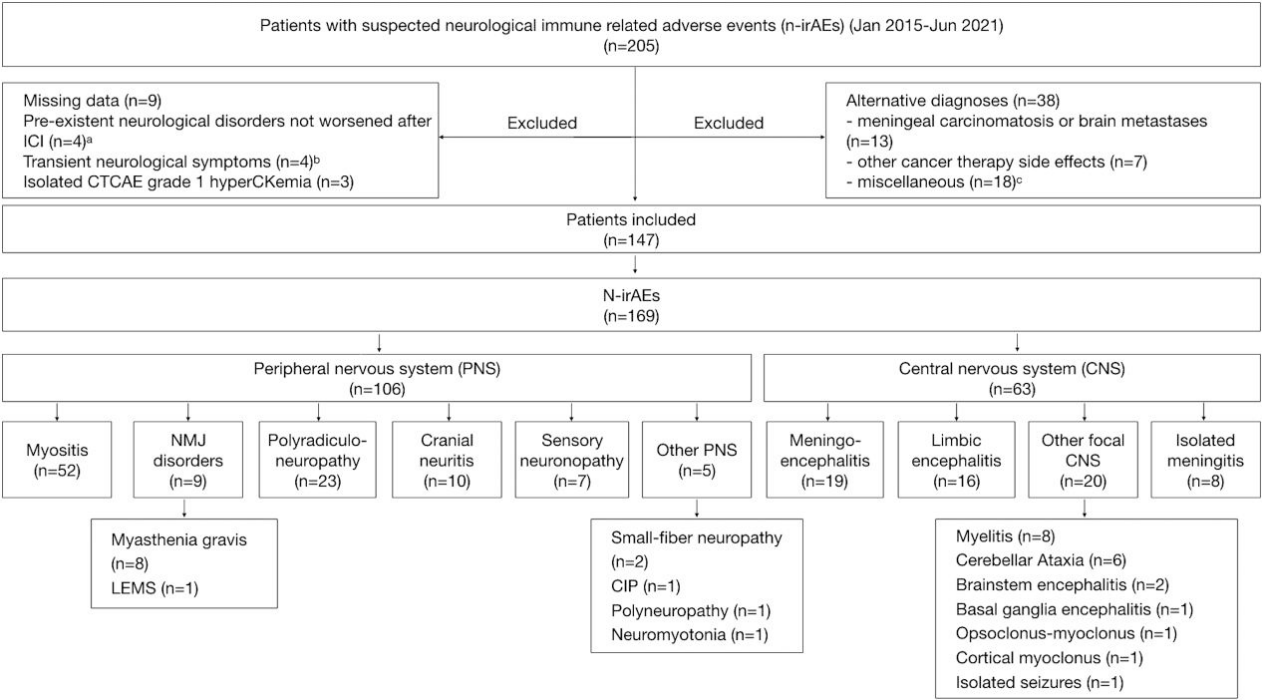
**Flow chart of patients indicating the observed clinical phenotypes.** ^a^γ-aminobutyric acid type B receptor- and Sox1-associated limbic encephalitis (*n* = 1), multifocal motor neuropathy (*n* = 1), critical illness neuropathy (*n* = 1), and seronegative rapidly progressive cerebellar ataxia (*n* = 1); ^b^Diplopia (*n* = 1), muscle weakness (*n* = 1), myalgias (*n* = 1) and paresthaesia (*n* = 1); ^c^Alcohol-related dementia (*n* = 2), Alzheimer disease (*n* = 2), arthralgias (*n* = 1), diabetes-related carpal tunnel syndrome (*n* = 1), clivus chondroma (*n* = 1), neurological functional disorder (*n* = 3), multifactorial gait disorder (*n* = 1), Parkinson disease (*n* = 1), primary headache (*n* = 1), sepsis-associated encephalopathy (*n* = 3) and steroid myopathy (*n* = 2). Abbreviations: CIP, chronic intestinal pseudo-obstruction; LEMS, Lambert–Eaton myasthenic syndrome; MG, myasthenia gravis; NMJ, neuromuscular junction; NMT, neuromyotonia.

A total of 147 patients were included (Lyon network: 84 patients; Paris network: 63 patients) out of 205 patients with a suspicion of n-irAE. N-irAEs involved the peripheral nervous system in 87/147 patients (59.2%), the CNS in 51/147 (34.7%), and both systems in 9/147 (6.1%) (Fig. 1, Supplementary Table 1). In all patients with CNS n-irAEs, cerebrospinal fluid analysis excluded the presence of malignant cells; brain MRI excluded the appearance of brain metastases in all but two patients (both myelitis) with CNS n-irAEs; previously known brain metastases in 13 patients had regressed (*n* = 8) or were stable (*n* = 5) at the time of neurological toxicity, and in no case, the metastatic location was congruent with the syndrome. Spinal cord MRI excluded the presence of spinal cord metastases in 8/8 patients with myelitis. Of the 147 included patients, 31/147 (22%) were previously published in other case series.^20–26^

The median age was 65 years (range 20–87) and 87/147 patients (59.2%) were male. A total of 126/147 patients (85.7%) had only one identifiable phenotype, while combinations of two or three different presentations were observed in 21/147 patients (14.3%, combinations shown in Supplementary Fig. 1). The most frequent phenotypes were myositis (52/147, 35.4%), either isolated (44/52, 84.6%) or associated with neuromuscular junction (NMJ)-like manifestations (8/52, 15.4%: motor fluctuations in six patients, decremental response to repetitive nerve stimulation in one patient, LEMS in one patient), followed by polyradiculoneuropathy (23/147, 15.6%), meningoencephalitis (19/147, 12.9%), cranial neuritis (10/147, 6.8%), myelitis (8/15, 5.4%) and meningitis (8/15, 5.4%). Meningoencephalitis patients were characterized by altered mental status (19/19, 100%), clinical or radiological signs of meningitis (8/19, 42.1%), fever (6/19, 31.6%), bulbar symptoms (5/19, 26.3%), seizures (3/19, 15.8%), ataxia (3/19, 15.8%), aphasia (2/19, 10.5%) and did not fulfil the current criteria for autoimmune limbic encephalitis.^14^ Moreover, 30/147 patients (20.4%) had paraneoplastic-like syndromes (limbic encephalitis, 16/30; SNN, 7/30; rapidly progressive cerebellar syndrome 5/30; opsoclonus-myoclonus, 1/30; chronic intestinal pseudo-obstruction (CIP), 1/30; LEMS, 1/30, Fig. 1); among patients with paraneoplastic-like syndromes, 2/30 (7%, both with SNN) the PNS was already diagnosed before the ICI treatment but they significantly worsened afterwards (mRS 3→4). Overall, neurological toxicity was an exacerbation of a pre-existent disease in 5/147 patients (3.4%), including SNN (*n* = 2), cortical myoclonus (*n* = 1), bilateral optic neuropathy (*n* = 1) and myositis (*n* = 1). There were two additional cases with previous myasthenia gravis (MG) that developed myositis after ICI initiation. Two patients (1.4%) had severe disability (mRS > 2) before the onset of n-irAE, due either to bone fractures in the inferior limb (*n* = 1) or to paraneoplastic cortical myoclonus (*n* = 1). Neurological toxicity arose after a median delay of 42 days (range 1–681, Supplementary Fig. 2) and two cycles (range 1–48) after ICI initiation and was acute or subacute in 142/147 patients (96.6%). Concomitant non-neurological irAEs were reported in 65/147 patients (44.2%) (Supplementary Table 1) and were less frequent in the case of paraneoplastic-like syndrome (6/29, 20.7%) compared to other phenotypes (59/118, 50.0%; *P* < 0.01). Anti-neural/glial antibodies were detected in 47/73 tested patients (64.4%) with diverse clinical presentations. Among them, paraneoplastic-related antibodies were detected in 24/73 patients (32.9%; Ma2, nine patients; Hu, seven patients; SOX1 and Yo, three patients each; CV2/CRMP5 and Ri, one patient each), including 19 patients (79.2%) with paraneoplastic-like syndromes. Other antibodies included anti-GFAP (six patients), CASPR2 (two patients), AGO2, TRIM9, and GAD65 antibodies (one patient each) and neuropil antibodies of unknown significance (12 patients, Supplementary Fig. 3). The serum of two patients was sampled before ICI administration (both positive for anti-neural antibodies: Ma2, *n* = 1; Yo, *n* = 1). Anti-acetylcholine receptor antibodies were detected in 11/27 (40.7%) tested cases, all with myositis/NMJ disorders.

### Cancer associations and immune checkpoint inhibitors regimens

**Figure 2:**
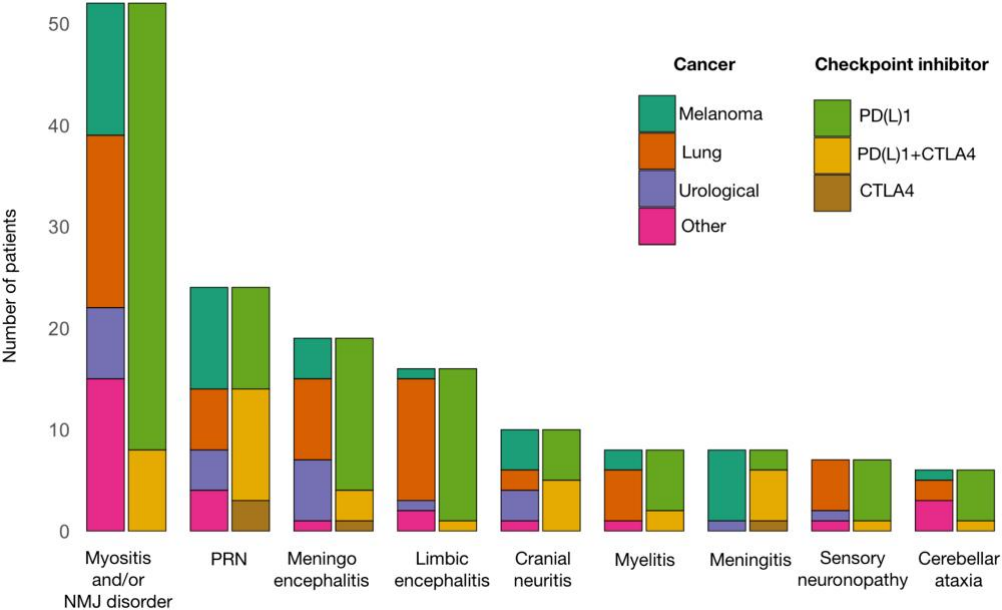
**Clinical presentations according to the types of ICIs and cancer**. Bar chart indicating the number of patients according to the types of cancer and ICI for each clinical phenotype. Anti-CTLA4 immunotherapy was more frequent in patients with polyradiculoneuropathy or meningitis, and less frequent in patients with myositis and/or MG, or limbic encephalitis. Conversely, melanoma was more frequent in meningitis patients, and lung cancer was more frequent in limbic encephalitis patients. Cancers other than melanoma, lung and urological cancer included thymoma (*n* = 4), Hodgkin lymphoma (*n* = 3), colorectal adenocarcinoma (*n* = 3), skin squamous cell carcinoma (*n* = 3), prostate adenocarcinoma (*n* = 2), Merkel carcinoma (*n* = 2), oropharynx squamous cell carcinoma (*n* = 1), breast adenocarcinoma (*n* = 1), pleural mesothelioma (*n* = 1), chordoma (*n* = 1), ovary carcinoma (*n* = 1), leiomyosarcoma (*n* = 1), acute myeloblastic leukaemia (*n* = 1), liposarcoma (*n* = 1) and hepatocellular carcinoma (*n* = 1). Abbreviations: PRN = polyradiculoneuropathy; NMJ = neuromuscular junction.

Malignancies included lung cancer (53/147, 36.1%, including 11/53, 20.8% patients with small-cell lung cancer), melanoma (45/147, 30.6%), urological cancers (23/147, 15.6%), and others (26/147, 17.7%; Supplementary Table 2). At the time of ICI introduction, cancer was metastatic (111/143, 77.6%) or locally advanced (32/143, 22.4%) in all cases for which the information was available, and 21/143 patients (14.7%) had brain metastases. Clinical phenotypes in patients with brain metastases included meningoencephalitis (isolated, *n* = 5; with cranial neuritis or myelitis, one patient each), limbic encephalitis (isolated, *n* = 5; with polyradiculoneuropathy, *n* = 1), myositis (isolated, *n* = 3; with NMJ disorder, *n* = 1), polyradiculoneuropathy (*n* = 2), myelitis (*n* = 1), opsoclonus-myoclonus (*n* = 1), small-fiber neuropathy (*n* = 1) and CIP (*n* = 1). ICI regimens included programmed cell death protein (ligan) 1 (PD(L)1) inhibitors (103/147, 70.1%), CTLA4 inhibitors (5/147, 3.4%), or combinations thereof (38/147, 25.9%); information was blinded for one patient (anti-PD1 alone or combined anti-PD1 and anti-CTLA4 therapy; Supplementary Table 3). Clinical phenotypes were not evenly distributed among cancer types and ICI: meningitis was more frequent among melanoma patients (7/45, 15.6%) compared to non-melanoma patients (1/102, 1.0%; *P* = 0.003), while paraneoplastic-like syndromes were more frequent among patients with lung cancer compared with other malignancies (21/53, 39.6% versus 9/94, 9.6%; *P* < 0.001). Regarding the type of ICI used, patients treated with anti-PD(L)1 alone were more in proportion to have myositis (35/103, 34%, versus 8/43, 18.6%; *P* = 0.007) or limbic encephalitis (15/103, 14.6%; versus 1/43, 2.3%; *P* = 0.04), while patients treated with anti-CTLA4 (alone or combined with anti-PD(L)1) were more in proportion to have polyradiculoneuropathy (14/43, 32.6% versus 9/103, 8.7%; *P* < 0.001) or meningitis (6/43, 14%; versus 2/101, 2%; *P* = 0.008; Fig. 2).

### Management, mortality, and outcomes

ICIs were discontinued due to n-irAE in 146/147 patients (99.5%), and the median delay from the first ICI dose to the last ICI dose was 40 days (range 0–760). Cancer progression was documented after ICI withdrawal in 69/135 cases (51.1%), after a median delay since the last ICI dose of 6 months (range 0.5–39). A total of 17 patients (11.5%) were later rechallenged with ICI and two of them had a neurological relapse. Overall, n-irAEs relapses were observed in 12 patients (8.2%), all were similar to the first event and occurred after a median delay of six (range 2–16) months after n-irAE onset (Supplementary Table 4).

Immune-active treatments were administered to 138/144 patients (95.8%), after a median delay of 12 days (range 0–371) from n-irAE onset. Steroids were administered in 134/144 patients (93%) and were the only immune-active treatment in 56/144 patients (38.9%). Other treatments included intravenous immunoglobulins (IVGs) (53/144, 36.8%), plasma exchange (22/144, 15.3%), and/or biotherapies and immunosuppressors (36/144, 25%, Table 1). Patients with paraneoplastic-like presentations tended to receive biotherapies and/or immunosuppressors more frequently than the rest of the cohort (10/27, 37% versus 24/117, 20.5%, *P* = 0.08).

**Table 1:** Treatment, cancer progression, ICI rechallenge and relapses according to the clinical presentation

	Myositis and/or NMJ disorders, *n* = 53	Polyradiculoneuropathy, *n* = 23	Sensory neuronopathy, *n* = 7	Cranial neuritis, *n* = 10	Meningoencephalitis, *n* = 19	Limbic encephalitis, *n* = 16	Myelitis, *n* = 8	Cerebellar ataxia, *n* = 6	Isolated meningitis, *n* = 8
**ICI discontinuation, *n/N* (%)**	53/53 (100)	23/23 (100)	7/7 (100)	10/10 (100)	18/19 (94.7)	16/16 (100)	8/8 (100)	6/6 (100)	8/8 (100)
**Immunological treatments, *n/N* (%)**									
CCS	50/52 (96.2)	21/23 (91.3)	6/6 (100)	9/10 (90)	16/19 (82.6)	14/14 (100)	8/8 (100)	6/6 (100)	7/8 (87.5)
IVIG	10/52 (19.2)	15/23 (65.2)	6/6 (100)	3/10 (10)	5/19 (26.3)	9/14 (64.3)	3/8 (37.5)	4/6 (66.7)	1/8 (12.5)
PEX	9/52 (17.3)	3/23 (13.1)	1/6 (16.7)	1/10 (10)	2/19 (10.5)	2/14 (14.3)	4/8 (50)	0 (0)	0/8 (0)
RTX	0/52 (0)	0/23 (0)	1/6 (16.7)	1/10 (10)	0/19 (0)	5/14 (35.7)	0/8 (0)	2 (33.3)	0/8 (0)
CYC	0/52 (0)	1/23 (4.3)	2/6 (33.3)	0/10 (0)	0/19 (0)	1/14 (7.1)	2/8 (25)	1 (16.7)	0/8 (0)
RUXO	9/52 (17.3)	0/23 (0)	0/6 (0)	0/10 (0)	0/19 (0)	0/14 (0)	1/8 (12.5)	0 (0)	0/8 (0)
**Median (range) follow-up duration, months**	11 (0.5–50)	7 (0.5–38)	14 (0.5–35)	17.5 (3–38)	13 (0.5–41)	5.5 (0.5–31)	14 (2–21)	6.5 (0.5–47)	27 (18.7–31.2)
**Cancer progression, *n/N* (%)**	24/46 (52.2)	10/20 (50)	3/7 (42.9)	3/10 (30)	9/19 (47.4)	7/14 (50.5)	3/8 (37.5)	2/6 (33.3)	4/8 (50)
**ICI rechallenge, *n/N* (%)**	4/46 (8.7)	5/20 (25)	1/6 (16.7)	2/10 (20)	3/18 (16.7)	0/15 (0)	0/8 (0)	0/5 (0)	2/8 (25)
**n-irAE relapse, *n/N* (%)**	1/47 (2.1)	3/22 (13.6)	0/6 (0)	0/10 (10)	2/18 (11.1)	2/13 (16.7)	2/8 (25)	1/5 (20)	0/8 (0)

Less frequent phenotypes (small-fiber neuropathy, *n* = 2, length-dependent polyneuropathy, *n* = 1, CIP, *n* = 1, neuromyotonia, *n* = 1) are not represented.

Abbreviations: CCS, corticosteroids; CYC, cyclophosphamide; IVIG, intravenous immunoglobulins; NMJ, neuromuscular junction; PEX, plasma exchange; RTX, rituximab; RUXO, ruxolitinib.

**Figure 3:**
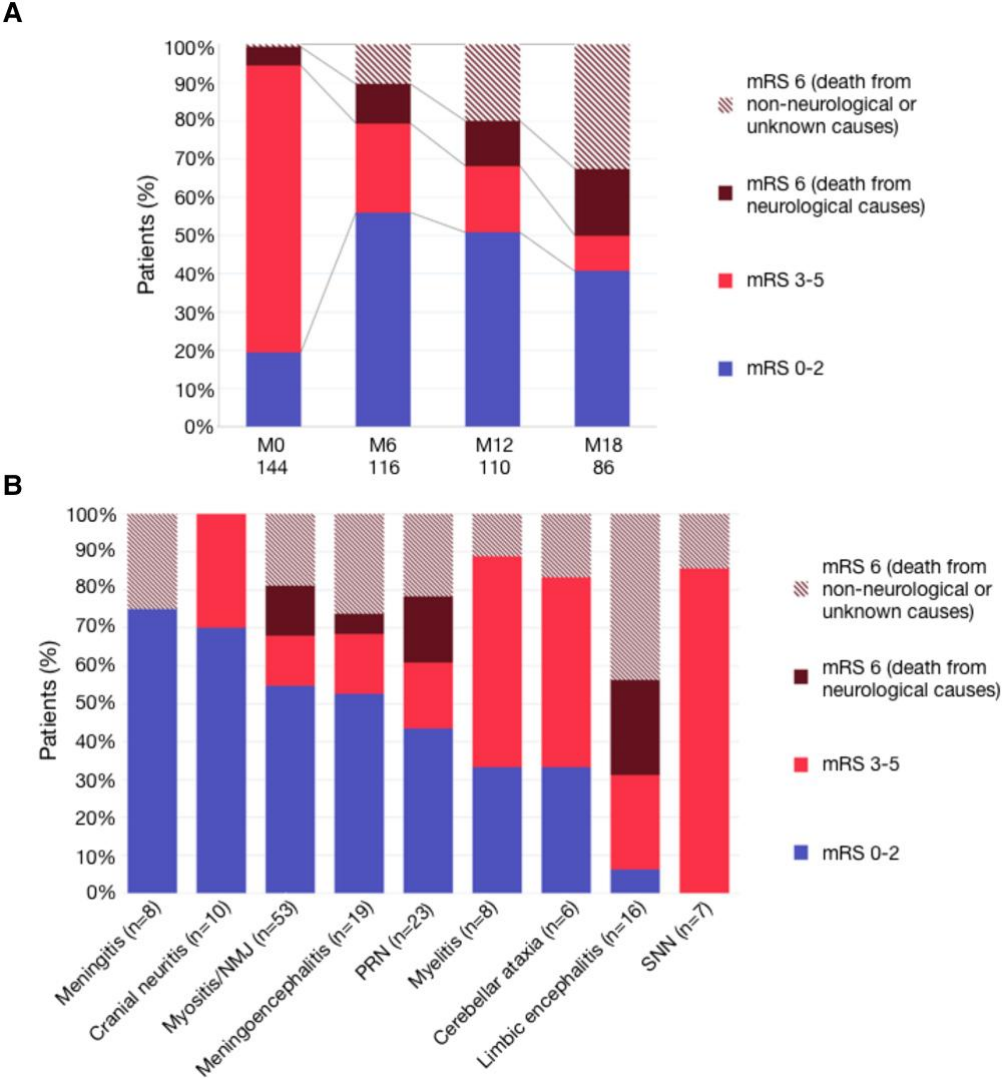
**Outcomes.** (**A**) Disability at onset (M0), 6 months (M6), 12 months (M12) and 18 months (M18). The proportion of patients with minor disability changed from 28/144 (19.4%) patients at onset to 65/116 (56.0%) at 6 months, 56/110 (50.9%) at 12 months, and 35/86 (40.7%) at 18 months, although mortality increased at all time points. (**B**) Outcome at last visit [median (range) follow-up duration: 12, (0.5–50) months] according to the initial clinical presentation. Less frequent phenotypes (small-fiber neuropathy, *n* = 2, length-dependent polyneuropathy, *n* = 1, CIP, *n* = 1, neuromyotonia, *n* = 1) are not represented. Abbreviations: mRS = modified Rankin score; NMJ = neuromuscular junction, PRN = polyradiculoneuropathy; SNN = sensory neuronopathy.

The median follow-up duration was 12 months (range 0.5–50). Severe disability (mRS 3-5) was reported in 108/144 patients (75.0%) at onset (onset mRS unknown for three patients), and 33/146 patients (22.6%) at the last visit. Autonomy for instrumental (CTCAE 1) or self-care (CTCAE ≤2) activities of daily life were preserved, respectively, in 49/113 (43.4%) and 65/113 patients (57.5%) at the last visit (Supplementary Fig. 4). The proportion of patients with minor disability changed from 28/144 (19.4%) at onset to 65/116 (56.0%) at 6 months, 56/110 (50.9%) at 12 months, and 35/86 (40.7%) at 18 months. Mortality rates increased gradually at 6, 12 and 18 months of follow-up (Fig. 3A), and a total of 48/147 patients (32.7%) died during follow-up (median delay from n-irAE onset to death: 6 months, range 0–26). The main causes of death were cancer progression (17/48, 35.4%), neurological toxicity (15/48, 31.2%), other causes (10/48, 20.8%) or unknown causes (6/48, 12.5%). The most frequent cause of death was neurological toxicity in the first 3 months after n-irAE onset (9/14, 64.3%), and cancer progression after 3 months (16/34 cases, 47.1%). Fatal n-irAEs included myositis (7/15, 46.7%, of which five with concomitant myocarditis), limbic encephalitis (4/15), polyradiculoneuropathy (4/15, including two cases associated with limbic and brainstem encephalitis, respectively) and meningoencephalitis (1/15). At the last visit (median 14 months, range 0.5–50), 65/98 surviving patients (66.3%) had a minor disability, although outcomes varied according to the phenotype: minor disability was frequent in patients with myositis/NMJ disorders, polyradiculoneuropathy, cranial neuritis, meningoencephalitis, and isolated meningitis, while most patients with myelitis, cerebellar ataxia, SNN and limbic encephalitis were severely disabled at last visit (Fig. 3B, Supplementary Fig. 5).

### Prognostic factors

**Figure 4:**
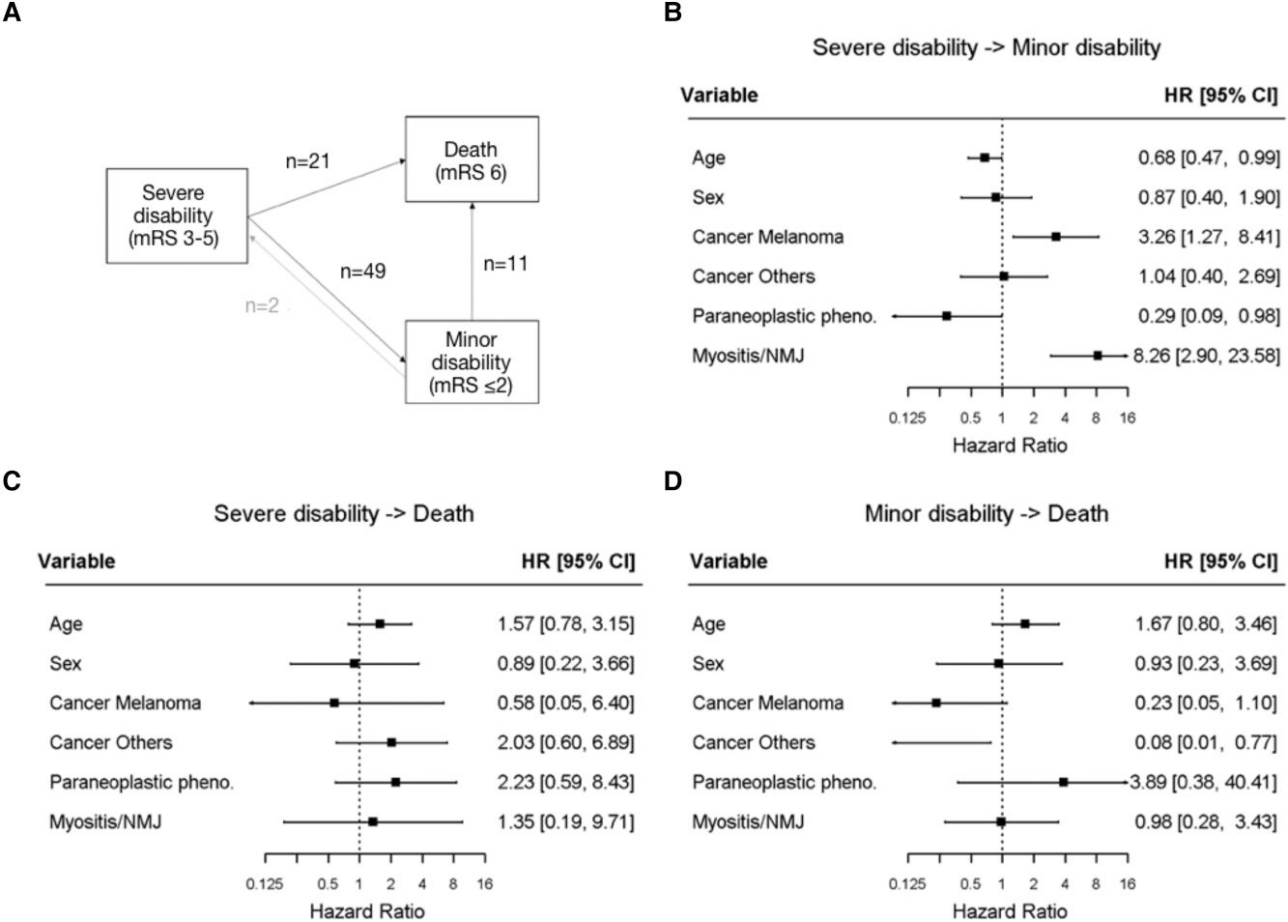
**Presentation of the multi-state Markov model and multivariate analysis of outcomes**. Each patient was categorized into one of three different states of disease at onset, 6 months, 12 months and 18 months: minor disability (mRS ≤2), severe disability (mRS 3-5), and death (mRS = 6); the transition rates from severe disability to a minor disability, severe disability to death and minor disability to death across these time points were estimated using maximum likelihood (**A**). Exposures were introduced into the different transitions to study their effects; forest plots showing the hazard ratios and confidence intervals according to the multivariate analysis for transition rates from severe disability to minor disability (**B**), severe disability to death (**C**) and minor disability to death (**D**), are represented. Melanoma and myositis/NMJ disorders were independently associated with a transition from severe to minor disability while increasing age and paraneoplastic-like syndromes independently decreased the rate of transition from severe to minor disability. Meanwhile, the transition rate from minor disability to death was decreased in patients with cancers other than lung and melanoma, compared to lung cancer. Abbreviations: CI = confidence interval; HR = hazard ratio; mRS = modified Rankin scale; NMJ = neuromuscular junction.

Figure 4A shows the transitions between severe disability, minor disability and death at the following time points: baseline, 6 months, 12 months, and 18 months. The transition from minor to severe disability was observed only in two cases and was not included in the statistical model. In univariate analysis, the transition rate from severe to minor disability was reduced in case of CNS involvement [hazard ratio (HR) = 0.25, 95%CI (0.12; 0.49); *P* < 0.01] and in case of paraneoplastic-like syndromes [HR = 0.16, 95%CI (0.06; 0.47); *P* < 0.01], while the probability of death increased in case of paraneoplastic-like syndromes [HR = 7.52, 95%CI (1.58; 35.87); *P* = 0.01], or presence of paraneoplastic-related antibodies [HR = 92.01, 95%CI (2.41; 3505.58); *P* = 0.01]. By contrast, myositis/NMJ disorder [HR = 5.02, 95%CI (2.55; 9.87); *P* < 0.01], melanoma [compared to lung cancer, HR = 2.92, 95%CI (1.48; 5.74); *P* < 0.01], concomitant CTLA4 and PD(L)1 therapy [HR = 2.36, 95%CI (1.23; 4.51); *P* < 0.01], and presence of non-neurological irAEs [HR = 1.81, 95%CI (1.02; 3.24); *P* = 0.04] were associated with an increased rate of transition from severe to minor disability (Supplementary Fig. 6). Age, sex, cancer category, presence of myositis/NMJ disorder and presence of a paraneoplastic-like syndrome were included in the multivariate analysis. In this model, older age [HR = 0.68, 95%CI (0.47; 0.99); *P* = 0.042] and paraneoplastic-like syndromes [HR = 0.29, 95%CI (0.09; 0.98); *P* = 0.047] independently decreased the transition rate from severe to minor disability. Conversely, melanoma [compared to lung cancer, HR = 3.26, 95%CI (1.27; 8.41); *P* = 0.01] and myositis/NMJ disorders [HR = 8.26, 95%CI (2.90; 23.58); *P* = 0.0001] independently increased the transition rate from severe to minor disability, while cancers other than lung and melanoma independently decreased the probability of transitioning from minor disability to death [HR = 0.08, 95%CI (0.01; 0.77); *P* = 0.03; Fig. 4B–D]. Of note, the rate of cancer progression was not increased in patients with a severe disability who did not transition towards minor disability, compared to the rest of the cohort (28/53, 52.8%, versus 41/82, 50.0%, *P* = 0.86).

## Discussion

The present study assessed the outcomes of n-irAEs patients referred to two expert networks in France over 5 years, using a multi-state illness-death model. The findings suggest that the probability of clinical improvement of n-irAE patients depends mainly on the clinical phenotype at presentation, age, and underlying cancer.

Although some n-irAEs may be successfully treated with steroids, reports regarding the neurological outcomes of these heterogeneous complications are conflicting, likely because of the small size of previously published cohorts.^8,25,26^ The present cohort is the largest reported yet and found that neurological recovery occurs in about half of the cases, even though a third of the patients died during the study period, mostly from tumour progression and neurological toxicity.

Strikingly, a fifth of the patients presented clinical phenotypes reminiscent of PNS, such as limbic encephalitis, rapidly progressive cerebellar ataxia or SNN. Patients with paraneoplastic-like n-irAEs were not clinically different from classical PNS, except that they had advanced-stage cancer, while classical PNS usually antedate the diagnosis of limited-stage cancer.^27^ In addition, paraneoplastic-like patients often had paraneoplastic-related antibodies, such as anti-Hu, anti-Ri, anti-Yo, and anti-Ma2 antibodies, which were also detected in pre-therapeutic samples in a few cases, in line with previous reports.^21,28–32^ Importantly, paraneoplastic-like syndromes, whether associated with paraneoplastic-related antibodies or not, independently decreased the probability of neurological recovery. The effects of paraneoplastic-like phenotypes and age on the transition rate from severe to minor disability will need confirmation (HR confidence interval upper limits approaching 1 in both cases) but is congruent with previous reports.^21,24,26^ Previous experimental data have shown that ICIs can trigger paraneoplastic-like neurological phenotypes in mice,^33^ and there have been reports of new-onset paraneoplastic-like syndromes, or exacerbations of previous PNS in ICIs-treated patients,^21,26,34^ suggesting an overlap between PNS and n-irAEs. The present data not only confirmed that n-irAE can present as paraneoplastic-like syndromes, but also emphasized important differences compared to other n-irAEs, notably their poorer prognosis (despite a trend towards more frequent use of biotherapies and/or immunosuppressors), more frequent association with paraneoplastic-related antibodies, and lower association with non-neurological irAE. Furthermore, the observed clinical differences might reflect distinct pathogenic mechanisms: for instance, paraneoplastic-like n-irAEs may result from the cross-reactivity of immune responses against neural antigens ectopically expressed in tumour cells (as described in classical PNS), whereas other n-irAEs might derive from cancer-independent autoimmune responses in predisposed individuals.^35^ Although paraneoplastic-related antibodies, such as anti-Hu, -Ma2 or -Yo antibodies, may indicate more severe presentations and/or distinct pathophysiological mechanisms,^36,37^ the relation between brain-reactive antibodies, ICI treatment, and the development of neurotoxicity is still not completely clarified. For instance, paraneoplastic-related and other brain-reactive antibodies can be detected in cancer patients without paraneoplastic neurological disorders or ICI treatment,^27,38^ while ICIs can trigger the production of serum brain-reactive antibodies even in the absence of neurotoxicity.^38^ In addition, findings in the literature regarding the frequency of brain-reactive antibodies are conflicting, especially in patients with ICI-induced encephalitis, possibly reflecting a referral bias towards paraneoplastic-like phenotypes in this and other studies and case reports.^26,36,37^ Prospective studies are therefore needed to clarify the factors leading to the different n-irAE phenotypes, and especially the role of brain-reactive antibodies.

Importantly, ∼1 in 7 of myositis/NMJ patients died due to neurological toxicity, indicating that ICI-induced myositis/NMJ disorder carries a high-risk of fatality, as outlined in previous studies.^9,39^ Nevertheless, among myositis/NMJ patients who survived the acute phase, the risk of long-term neurological disability was relatively low (around 10%), in line with previous studies showing clinical improvement in about 70% of myositis patients.^40,41^ This is in contrast with patients with paraneoplastic-like presentations, who were less likely to achieve neurological recovery. MG was found in addition to myositis in 15% of ICIs-related patients, and isolated in one case, as previously reported.^42^ Of note, NMJ malfunction was documented electrically in only one of these patients, while the presence of acetylcholine receptor antibodies in myositis patients was not correlated with any clinical or electrical evidence of NMJ disorder, as suggested by previous reports.^42^ Considering the peculiar distribution of muscle deficits (i.e. oculomotor and orbicular muscles, and bulbar and respiratory muscles), ICI-related myositis may be confused with MG; hence, some authors recommend considering the diagnostic of MG only when decremental responses are obtained after repetitive nerve stimulation.^12^

In the present series, older age was independently associated with lesser chances of recovery, confirming previous observations.^26^ In addition, in line with previous studies,^26^ co-occurring non-neurological irAEs were associated with neurological recovery, likely because they were less frequent in patients with paraneoplastic-like syndromes. Remarkably, melanoma was an independent predictor of neurological recovery, probably because the most frequent phenotypes in the melanoma patients herein (i.e. polyradiculoneuropathy and meningitis) had high recovery rates, in opposition to other phenotypes more frequently found in lung cancer patients (i.e. paraneoplastic-like syndromes such as limbic encephalitis and SNN). It is noteworthy that polyradiculoneuropathy and meningitis were also more frequently observed in patients with anti-CTLA4 treatments, indicating that the type of ICI may also influence the type of clinical presentation and therefore the outcomes. These findings are consistent with previous studies^9^ and highlight the need for large-scale, prospective studies to disentangle the respective roles of cancer type and immunotherapy in the determination of neurological phenotypes and outcomes.

About a third of the patients studied herein died over the study period, consistently with previously reported mortality rates.^25^ Half the patients experienced cancer progression, which, in line with previous findings,^8^ was the main identified cause of death, and lung cancer carried the highest risk of fatal outcome. It is unclear whether ICI withdrawal, steroids, and other immunosuppressants foster cancer progression,^8^ but considering the lack of alternative oncological treatment options, the question of ICI rechallenge is crucial for these patients. Importantly, the incidence of neurological relapses among patients who were ICI-rechallenged was low (around 12%) in the present cohort compared to others (60%,^8^ possibly because of wider use of immunosuppressants), including in the 17 patients who were ICI-rechallenged. Further studies are needed to determine whether ICI rechallenge is safe and effective in patients with n-irAE once sustained neurological improvement is obtained.^43–45^

Limitations of this study include the variability of follow-up durations and the fact that we simultaneously assessed two competing risks (neurological disability and death); we overcame these issues by using a multi-state statistical model. Other limitations include the retrospective design of the study, with the possible occurrence of misdiagnoses considering the absence of validated clinical criteria for n-irAEs, the heterogeneity of data sources, the imbalance of sample size in the different phenotype categories, the limited number of factors assessed in the multivariate analysis, and a referral bias towards severe presentations and paraneoplastic-like phenotypes, with a possible impact on the phenotypic profiles and outcomes. Due to the retrospective data collection, it was also not always possible to accurately discriminate factors associated with death from neurotoxicity compared to other causes, and therefore the cause of death was unknown in a substantial proportion of cases. Conversely, cancer progression is unlikely to have influenced the assessed level of long-term disability as cancer progression rates were not higher in patients who did not improve their mRS score. Prospective studies are needed in order to overcome the limitations of the present retrospective study and to confirm its findings.

In conclusion, the present data showed that neurological recovery in n-irAE patients depends on the baseline characteristics and the type of clinical presentation and that myositis/NMJ disorders and melanoma are associated with a higher probability of improvement, while older age and paraneoplastic-like syndromes are associated with lower chances of neurological recovery. Future studies are needed to optimize the management of such patients.

## Supplementary Material

fcad169_Supplementary_Data

## Data Availability

Data reported in this study are available within the article and/or its Supplementary material. More information is available from the corresponding author on reasonable request.

## Abbreviations

CI =: confidence interval
CTCAE =: common terminology criteria for adverse events
ENMG =: electroneuromyography
HR =: hazard ratio
ICIs =: immune checkpoint inhibitors
irAEs =: immune-related adverse events
MG =: myasthenia gravis
mRS =: modified Rankin scale
n-irAEs =: neurological immune-related adverse events
NMJ =: neuromuscular junction
PNS =: paraneoplastic neurological syndromes

## References

[1] Martins F , Sykiotis GP, Maillard M, et al New therapeutic perspectives to manage refractory immune checkpoint-related toxicities. Lancet Oncol. 2019;20(1):e54–e64. 30614479 10.1016/S1470-2045(18)30828-3

[2] Robert C . A decade of immune-checkpoint inhibitors in cancer therapy. Nat Commun. 2020;11(1):3801. 32732879 10.1038/s41467-020-17670-y PMC7393098

[3] Ribas A , Wolchok JD. Cancer immunotherapy using checkpoint blockade. Science. 2018;359(6382):1350–1355. 29567705 10.1126/science.aar4060 PMC7391259

[4] Johnson DB , Manouchehri A, Haugh AM, et al Neurologic toxicity associated with immune checkpoint inhibitors: A pharmacovigilance study. J Immunotherapy Cancer. 2019;7(1):134. 10.1186/s40425-019-0617-x PMC6530194 31118078

[5] Sato K , Mano T, Iwata A, Toda T. Neurological and related adverse events in immune checkpoint inhibitors: A pharmacovigilance study from the Japanese adverse drug event report database. J Neurooncol. 2019;145(1):1–9. 31452071 10.1007/s11060-019-03273-1

[6] Larkin J , Chmielowski B, Lao CD, et al Neurologic serious adverse events associated with nivolumab plus ipilimumab or nivolumab alone in advanced melanoma, including a case series of encephalitis. Oncologist. 2017;22(6):709–718. 28495807 10.1634/theoncologist.2016-0487 PMC5469590

[7] Kao JC , Brickshawana A, Liewluck T. Neuromuscular complications of programmed cell death-1 (PD-1) inhibitors. Curr Neurol Neurosci Rep. 2018;18(10):63. 30078154 10.1007/s11910-018-0878-7

[8] Dubey D , David WS, Reynolds KL, et al Severe neurological toxicity of immune checkpoint inhibitors: Growing spectrum. Ann Neurol. 2020;87(5):659–669. 32086972 10.1002/ana.25708

[9] Marini A , Bernardini A, Gigli GL, et al Neurologic adverse events of immune checkpoint inhibitors: A systematic review. Neurology. 2021;96(16):754–766. 33653902 10.1212/WNL.0000000000011795

[10] Wang DY , Salem JE, Cohen JV, et al Fatal toxic effects associated with immune checkpoint inhibitors: A systematic review and meta-analysis. JAMA Oncol. 2018;4(12):1721. 30242316 10.1001/jamaoncol.2018.3923 PMC6440712

[11] Esfahani K , Elkrief A, Calabrese C, et al Moving towards personalized treatments of immune-related adverse events. Nat Rev Clin Oncol. 2020;17(8):504–515. 32246128 10.1038/s41571-020-0352-8

[12] Guidon AC , Burton LB, Chwalisz BK, et al Consensus disease definitions for neurologic immune-related adverse events of immune checkpoint inhibitors. J Immunother Cancer. 2021;9(7):e002890. 10.1136/jitc-2021-002890 PMC8291304 34281989

[13] Graus F , Vogrig A, Muñiz-Castrillo S, et al Updated diagnostic criteria for paraneoplastic neurologic syndromes. Neurol Neuroimmunol Neuroinflamm. 2021;8(4):e1014. 10.1212/NXI.0000000000001014 PMC8237398 34006622

[14] Graus PF , Titulaer MJ, Balu R, et al A clinical approach to diagnosis of autoimmune encephalitis. Lancet Neurol. 2016;15(4):391. 26906964 10.1016/S1474-4422(15)00401-9 PMC5066574

[15] Hébert J , Riche B, Vogrig A, et al Epidemiology of paraneoplastic neurologic syndromes and autoimmune encephalitides in France. Neurol Neuroimmunol Neuroinflamm. 2020;7(6):e883. 32847939 10.1212/NXI.0000000000000883 PMC7455315

[16] Graus F , Keime-Guibert F, Reñe R, et al Anti-Hu-associated paraneoplastic encephalomyelitis: Analysis of 200 patients. Brain. 2001;124(Pt 6):1138–1148. 11353730 10.1093/brain/124.6.1138

[17] Brahmer JR , Lacchetti C, Schneider BJ, et al Management of immune-related adverse events in patients treated with immune checkpoint inhibitor therapy: American Society of Clinical Oncology Clinical Practice Guideline. J Clin Oncol. 2018;36(17):1714–1768. 29442540 10.1200/JCO.2017.77.6385 PMC6481621

[18] Anderson TW , Goodman LA. Statistical inference about markov chains. Annals Math Stat. 1957;28(1):89–110.

[19] Jackson C . Multi-State models for panel data: The MSM package for R. J Stat Softw. 2011;38:1–28.

[20] Vogrig A , Muñiz-Castrillo S, Joubert B, et al Central nervous system complications associated with immune checkpoint inhibitors. J Neurol Neurosurg Psychiatry. 2020;91(7):772–778. 32312871 10.1136/jnnp-2020-323055

[21] Vogrig A , Fouret M, Joubert B, et al Increased frequency of anti-Ma2 encephalitis associated with immune checkpoint inhibitors. Neurol Neuroimmunol Neuroinflamm. 2019;6(6):e604. 31454760 10.1212/NXI.0000000000000604 PMC6705619

[22] Mongay-Ochoa N , Vogrig A, Muñiz-Castrillo S, Honnorat J. Anti-Hu-associated paraneoplastic syndromes triggered by immune-checkpoint inhibitor treatment. J Neurol. 2020;267(7):2154–2156. 32451614 10.1007/s00415-020-09940-y

[23] Vogrig A , Muñiz-Castrillo S, Joubert B, et al Cranial nerve disorders associated with immune checkpoint inhibitors. Neurology. 2021;96 (6):e866–e875 33318162 10.1212/WNL.0000000000011340

[24] Farina A , Villagrán-García M, Ciano-Petersen NL, et al Anti-Hu antibodies in patients with neurologic Side effects of immune checkpoint inhibitors. Neurol Neuroimmunol Neuroinflamm. 2023;10(1):e200058. 10.1212/NXI.0000000000200058 PMC9709718 36446613

[25] Plaçais L , Michot JM, Champiat S, et al Neurological complications induced by immune checkpoint inhibitors: A comprehensive descriptive case-series unraveling high risk of long-term sequelae. Brain Commun. 2021;3(4):fcab220. 10.1093/braincomms/fcab220 PMC8505025 34651126

[26] Sechi E , Markovic SN, McKeon A, et al Neurologic autoimmunity and immune checkpoint inhibitors: Autoantibody profiles and outcomes. Neurology. 2020;95(17):e2442–e2452. 32796130 10.1212/WNL.0000000000010632 PMC7682911

[27] Graus F , Dalmau J. Paraneoplastic neurological syndromes in the era of immune-checkpoint inhibitors. Nat Rev Clin Oncol. 2019;16(9):535–548. 30867573 10.1038/s41571-019-0194-4

[28] Shibaki R , Murakami S, Oki K, Ohe Y. Nivolumab-induced autoimmune encephalitis in an anti-neuronal autoantibody-positive patient. Jpn J Clin Oncol. 2019;49(8):793–794. 31187869 10.1093/jjco/hyz087

[29] Albarrán V , Pozas J, Rodríguez F, et al Acute anti-Ma2 paraneoplastic encephalitis associated to pembrolizumab: A case report and review of literature. Transl Lung Cancer Res. 2021;10(7):3303–3311. 34430366 10.21037/tlcr-21-222 PMC8350103

[30] Papadopoulos KP , Romero RS, Gonzalez G, Dix JE, Lowy I, Fury M. Anti-Hu-associated autoimmune limbic encephalitis in a patient with PD-1 inhibitor-responsive myxoid chondrosarcoma. Oncologist. 2018;23(1):118–120. 29158368 10.1634/theoncologist.2017-0344 PMC5759826

[31] Matsuoka H , Kimura H, Koba H, et al Nivolumab-induced limbic encephalitis with anti-Hu antibody in a patient with advanced pleomorphic carcinoma of the lung. Clin Lung Cancer. 2018;19(5):e597–e599. 29857970 10.1016/j.cllc.2018.04.009

[32] Morimoto T , Orihashi T, Yamasaki K, Tahara M, Kato K, Yatera K. Paraneoplastic sensory polyneuropathy related to anti-PD-L1-including anticancer treatment in a patient with lung cancer. Intern Med. 2021;60(10):1577–1581. 33328400 10.2169/internalmedicine.5629-20 PMC8188014

[33] Yshii LM , Gebauer CM, Pignolet B, et al CTLA4 Blockade elicits paraneoplastic neurological disease in a mouse model. Brain. 2016;139(11):2923–2934. 27604307 10.1093/brain/aww225

[34] Manson G , Maria ATJ, Poizeau F, et al Worsening and newly diagnosed paraneoplastic syndromes following anti-PD-1 or anti-PD-L1 immunotherapies, a descriptive study. J Immunother Cancer. 2019;7(1):337. 31796119 10.1186/s40425-019-0821-8 PMC6892018

[35] Vogrig A , Muñiz-Castrillo S, Desestret V, Joubert B, Honnorat J. Pathophysiology of paraneoplastic and autoimmune encephalitis: Genes, infections, and checkpoint inhibitors. Ther Adv Neurol Disord. 2020;13:1756286420932797. 10.1177/1756286420932797 PMC7318829 32636932

[36] Velasco R , Villagrán M, Jové M, et al Encephalitis induced by immune checkpoint inhibitors: A systematic review. JAMA Neurol. 2021;78(7):864–873. 33720308 10.1001/jamaneurol.2021.0249

[37] Nersesjan V , McWilliam O, Krarup LH, Kondziella D. Autoimmune encephalitis related to cancer treatment with immune checkpoint inhibitors: A systematic review. Neurology. 2021;97(2):e191–e202. 33952651 10.1212/WNL.0000000000012122

[38] Müller-Jensen L , Knauss S, Ginesta Roque L, et al Autoantibody profiles in patients with immune checkpoint inhibitor-induced neurological immune related adverse events. Front Immunol. 2023;14:1108116. 10.3389/fimmu.2023.1108116 PMC9945255 36845122

[39] Boutros A , Bottini A, Rossi G, et al Neuromuscular and cardiac adverse events associated with immune checkpoint inhibitors: Pooled analysis of individual cases from multiple institutions and literature. ESMO Open. 2023;8(1):100791. 10.1016/j.esmoop.2023.100791 PMC9958259 36791639

[40] Touat M , Maisonobe T, Knauss S, et al Immune checkpoint inhibitor-related myositis and myocarditis in patients with cancer. Neurology. 2018;91(10):e985–e994. 30089619 10.1212/WNL.0000000000006124

[41] Shelly S , Triplett JD, Pinto MV, et al Immune checkpoint inhibitor-associated myopathy: A clinicoseropathologically distinct myopathy. Brain Commun. 2020;2(2):fcaa181. 10.1093/braincomms/fcaa181 PMC7713997 33305263

[42] Aldrich J , Pundole X, Tummala S, et al Inflammatory myositis in cancer patients receiving immune checkpoint inhibitors. Arthritis Rheumatol. 2021;73(5):866–874. 33258544 10.1002/art.41604

[43] Cuzzubbo S , Tetu P, Guegan S, et al Reintroduction of immune-checkpoint inhibitors after immune-related meningitis: A case series of melanoma patients. J Immunother Cancer. 2020;8(2):e001034. 10.1136/jitc-2020-001034 PMC7398097 32747471

[44] Weill A , Delyon J, Descamps V, et al Treatment strategies and safety of rechallenge in the setting of immune checkpoint inhibitors-related myositis: A national multicentre study. Rheumatology. 2021;60(12):5753–5764. 33725115 10.1093/rheumatology/keab249

[45] Villagrán-García M , Velasco R. Neurotoxicity and safety of the rechallenge of immune checkpoint inhibitors: A growing issue in neuro-oncology practice. Neurol Sci. 2022;43(4):2339–2361. 35175441 10.1007/s10072-022-05920-4

