# Supplementary material for: Neurological outcomes in immune checkpoint inhibitor-related neurotoxicity
Source: Brain Commun. 2023 May 27;5(3):fcad169. doi: 10.1093/braincomms/fcad169 (PMC10306160; doi:10.1093/braincomms/fcad169)
Supplement: fcad169_Supplementary_Data [file fcad169_supplementary_data.docx]

**SUPPLEMENTARY TABLES**

**Supplementary Table 1. Individual patients’ data of demographics, clinical variables, and outcomes.**

| **Patient number** | **Age (years),** | **Sex** | **Cancer type** | **Type of ICI** | **N-irAEs phenotype** | **Antineural antibodies** | **Non-neurological irAEs** | **mRS onset** | **mRS 6 months** | **mRS 12 months** | **mRS 18 months** | **Follow-up (months)** | **mRS last follow-up** |
| --- | --- | --- | --- | --- | --- | --- | --- | --- | --- | --- | --- | --- | --- |
| 1 | 32 | Male | Hodgkin lymphoma | Anti-PD(L)1 | Cerebellitis | Unknown antigen | None | 4 | 0 | 0 | 0 | 47 | 0 |
| 2 | 68 | Male | Lung adenocarcinoma | Anti-PD(L)1 | Limbic encephalitis | Ma2 | None | 4 | 6 |  |  | 1 | 6 |
| 3 | 72 | Female | Melanoma | Anti-CTLA4 | Basal ganglia encephalitis, polyradiculoneuropathy | CASPR2 | Colitis, thyroidits | 4 | 2 | NA | 4 | 26 | 6 |
| 4 | 47 | Male | Lung adenocarcinoma | Anti-PD(L)1 | Limbic encephalitis | Ma2 | None | 5 | 6 |  |  | 3 | 6 |
| 5 | 87 | Male | Merkel carcinoma | Anti-PD(L)1 | Polyneuropathy | Cv2 | None | 3 | 3 | 6 |  | 10 | 6 |
| 6 | 79 | Male | Lung adenocarcinoma | Anti-PD(L)1 | Limbic encephalitis | Ma2 | None | 5 | 2 | 6 |  | 9 | 6 |
| 7 | 57 | Female | Lung adenocarcinoma | Anti-PD(L)1 | Limbic encephalitis | Ma2 | None | 3 | 6 |  |  | 4 | 6 |
| 8 | 80 | Male | Melanoma | Anti-PD(L)1 | Limbic encephalitis | Unknown antigen | Vitiligo | 3 | 3 | 3 | 6 | 13 | 6 |
| 9 | 53 | Male | Renal clear cell carcinoma | Anti-PD(L)1 | Limbic encephalitis | Ma2 | None | 4 | 3 | 3 | NA | 31 | 4 |
| 10 | 76 | Male | Urothelial bladder carcinoma | Anti-PD(L)1 | Meningoencephalitis | Unknown antigen | Colitis | 4 | 6 |  |  | 1 | 6 |
| 11 | 79 | Male | Lung adenocarcinoma | Anti-PD(L)1 | Myositis | Negative | None | 3 | 2 | 2 | 2 | 50 | 1 |
| 12 | 46 | Female | Melanoma | Combined anti-PD(L)1 and anti-CTLA4 | Meningitis | Negative | Hypophysitis | 3 | 0 | 0 | 0 | 34 | 0 |
| 13 | 75 | Female | Melanoma | Combined anti-PD(L)1 and anti-CTLA4 | Cranial neuritis | Negative | None | 3 | 2 | 2 | 2 | 38 | 2 |
| 14 | 73 | Male | Lung adenocarcinoma | Anti-PD(L)1 | Myoclonus | Unknown antigen | None | 4 | 4 | 4 | 6 | 16 | 6 |
| 15 | 20 | Female | Melanoma | Combined anti-PD(L)1 and anti-CTLA4 | Myositis | Not tested | None | 2 | 0 | 0 | 0 | 35 | 0 |
| 16 | 65 | Male | Melanoma | Anti-CTLA4 | Polyradiculoneuropathy | Not tested | Diabetes, thyroiditis, hepatitis | 4 | 2 | 2 | 2 | 22 | 6 |
| 17 | 63 | Male | Melanoma | Combined anti-PD(L)1 and anti-CTLA4 | Cranial neuritis | Not tested | Thyroiditis | 2 | 0 | 0 | 0 | 38 | 0 |
| 18 | 62 | Male | Urothelial bladder carcinoma | Combined anti-PD(L)1 and anti-CTLA4 | Brainstem encephalitis | Negative | None | 2 | 0 | 0 | 0 | 35 | 0 |
| 19 | 73 | Male | Chordoma | Anti-PD(L)1 | Overlap Myositis-NMJ disorder (MG) | Not tested | None | 5 | 1 | 1 | 1 | 40 | 1 |
| 20 | 55 | Male | Squamous cell lung cancer | Anti-PD(L)1 | Meningoencephalitis, polyradiculoneuropathy | Negative | None | 5 | 4 | 4 | 4 | 38 | 4 |
| 21 | 56 | Female | Lung adenocarcinoma | Anti-PD(L)1 | Polyradiculoneuropathy | Not tested | None | 4 | 1 | 6 |  | 7 | 6 |
| 22 | 70 | Female | Lung adenocarcinoma | Anti-PD(L)1 | Meningoencephalitis | Negative | Thyroiditis | 4 | 0 | 0 | 6 | 17 | 6 |
| 23 | 63 | Male | Lung adenocarcinoma | Anti-PD(L)1 | Meningoencephalitis | GFAP | None | 5 | NA | NA | NA | 26 | 2 |
| 24 | 46 | Male | Small cell lung cancer | Anti-PD(L)1 | Sensory neuronopathy | Hu | None | 4 | 6 |  |  | 2 | 6 |
| 25 | 72 | Male | Lung adenocarcinoma | Anti-PD(L)1 | Opsoclonus myoclonus | Unknown antigen | Polyarthritis | 4 | NA | NA | 4 | 21 | 4 |
| 26 | 59 | Male | Lung adenocarcinoma | Anti-PD(L)1 | Meningoencephalitis, myelitis | GFAP | None | 5 | 2 | 2 | 2 | 21 | 2 |
| 27 | 68 | Male | Lung adenocarcinoma | Anti-PD(L)1 | Overlap Myositis-NMJ disorder (LEMS) | Sox1 | None | 4 | 2 | 6 |  | 8 | 6 |
| 28 | 36 | Female | Spleen lyposarcoma | Anti-PD(L)1 | Limbic encephalitis | Ma2 | None | 3 | 6 |  |  | 9 | 6 |
| 29 | 70 | Male | Small cell lung cancer | Anti-PD(L)1 | Cerebellitis | Hu | None | 4 | 3 | 4 | 5 | 18 | 5 |
| 30 | 82 | Female | Hodgkin lymphoma | Anti-PD(L)1 | Meningoencephalitis | Negative | None | 5 | 6 |  |  | 3 | 6 |
| 31 | 60 | Female | Lung adenocarcinoma | Anti-PD(L)1 | Myelitis | Yo | None | 4 | 4 | 4 |  | 14 | 5 |
| 32 | 65 | Female | Thymoma | Anti-PD(L)1 | Myositis, neuromyotonia | CASPR2 | None | 6 |  |  |  | 0.5 | 6 |
| 33 | 58 | Male | Small cell lung cancer | Anti-PD(L)1 | Limbic encephalitis | Sox1 | None | 3 | NA | 1 |  | 14 | 1 |
| 34 | 76 | Male | Renal clear cell carcinoma | Anti-PD(L)1 | Polyradiculoneuropathy, cranial neuritis | Negative | None | 2 | 1 | 0 |  | 9 | 0 |
| 35 | 86 | Female | Melanoma | Anti-PD(L)1 | Meningoencephalitis | Unknown antigen | None | 4 | NA | 2 |  | 8 | 2 |
| 36 | 55 | Male | Renal clear cell carcinoma | Anti-PD(L)1 | Meningoencephalitis | GFAP | None | 4 | NA | 1 |  | 14 | 1 |
| 37 | 57 | Male | Lung adenocarcinoma | Anti-PD(L)1 | Myelitis | Negative | None | 4 | 4 | 5 | 5 | 21 | 5 |
| 38 | 54 | Male | Renal clear cell carcinoma | Combined anti-PD(L)1 and anti-CTLA4 | Sensory neuronopathy | Negative | Thyroiditis | 4 | 3 | 3 |  | 14 | 4 |
| 39 | 70 | Male | Squamous cell lung cancer | Anti-PD(L)1 | Limbic encephalitis, sensory neuronopathy | AGO2 | None | 4 | 4 | 4 |  | 14 | 4 |
| 40 | 60 | Male | Lung adenocarcinoma | Anti-PD(L)1 | Cerebellitis | Yo | None | NA | 6 |  |  | 4 | 6 |
| 41 | 52 | Female | Ovarian clear cell carcinoma | Anti-PD(L)1 | Cerebellitis | Yo | None | 4 | 4 | 4 |  | 9 | 4 |
| 42 | 62 | Female | Lung adenocarcinoma | Anti-PD(L)1 | Limbic encephalitis | Ma2 | None | 4 | 3 | 6 |  | 5 | 6 |
| 43 | 72 | Male | Renal clear cell carcinoma | Combined anti-PD(L)1 and anti-CTLA4 | Meningoencephalitis, polyradiculoneuropathy | Unknown antigen | Nephritis | 2 | 1 | 1 |  | 9 | 1 |
| 44 | 56 | Male | Lung adenocarcinoma | Anti-PD(L)1 | Meningoencephalitis | GFAP | None | 2 | 1 |  |  | 7 | 1 |
| 45 | 65 | Male | Lung adenocarcinoma | Anti-PD(L)1 | Meningoencephalitis | Ri | Scleroderma, thrombocytopenia | 5 | NA | 4 |  | 13 | 4 |
| 46 | 78 | Male | Renal clear cell carcinoma | Anti-PD(L)1 | Cranial neuritis | Ma2 | None | 3 | 3 |  |  | 6 | 3 |
| 47 | 70 | Male | Mesothelioma | Combined anti-PD(L)1 and anti-CTLA4 | Limbic encephalitis | Ma2 | None | 3 | 6 |  |  | 6 | 6 |
| 48 | 64 | Female | Lung adenocarcinoma | Anti-PD(L)1 | Meningoencephalitis, Myelitis | GFAP | None | 4 |  |  |  | 2 | 2 |
| 49 | 66 | Female | Retroperitoneal leiomyosarcoma | Anti-PD(L)1 | Cerebellitis, myelitis, polyradiculoneuropathy | Negative | None | 4 |  |  |  | 3 | 3 |
| 50 | 68 | Female | Melanoma | Anti-PD(L)1 | Meningoencephalitis | Negative | Hypophysitis, polyarthritis | 4 |  |  |  | 3 | 0 |
| 51 | 48 | Female | Lung adenocarcinoma | Anti-PD(L)1 | CIP | Negative | Ileitis | 3 |  |  |  | 3 | 3 |
| 52 | 72 | Male | Renal clear cell carcinoma | Anti-PD(L)1 | Meningoencephalitis | GFAP | None | 5 | 4 | 4 | 6 | 15 | 6 |
| 53 | 74 | Female | Lung adenocarcinoma | Anti-PD(L)1 | Limbic encephalitis, sensory neuronopathy | Hu | None | 4 |  |  |  | 0.5 | 4 |
| 54 | 53 | Female | Small cell lung cancer | Anti-PD(L)1 | Meningoencephalitis, cranial neuritis | TRIM9 | None | 4 |  |  |  | 3 | 5 |
| 55 | 65 | Male | Small cell lung cancer | Anti-PD(L)1 | Limbic encephalitis, polyradiculoneuropathy | Hu | None | 4 | 6 |  |  | 3 | 6 |
| 56 | 60 | Female | Melanoma | Combined anti-PD(L)1 and anti-CTLA4 | Cranial neuritis | Negative | None | 2 | 2 | 2 | 2 | 20 | 2 |
| 57 | 63 | Male | Squamous cell lung cancer | Anti-PD(L)1 | Limbic encephalitis | Sox1 | Thyroiditis | 5 | 3 | 3 | 6 | 16 | 6 |
| 58 | 71 | Male | Melanoma | Anti-CTLA4 | Polyradiculoneuropathy | Negative | Polyarthritis, hepatitis | 4 | 1 |  |  | 7 | 1 |
| 59 | 59 | Female | Melanoma | Combined anti-PD(L)1 and anti-CTLA4 | Polyradiculoneuropathy | Not tested | None | 4 | 2 | 2 | 2 | 18 | 6 |
| 60 | 66 | Female | Melanoma | Anti-CTLA4 | Meningitis | Not tested | Hypophysitis | 5 | 1 | 2 | 2 | 19 | 6 |
| 61 | 65 | Female | Lung adenocarcinoma | Anti-PD(L)1 | Myositis | Not tested | Myocarditis | 4 | NA | 1 | 1 | 38 | 3 |
| 62 | 74 | Male | Lung adenocarcinoma | Anti-PD(L)1 | Overlap Myositis-NMJ disorder (MG) | Not tested | None | 4 | 3 | 6 |  | 6 | 6 |
| 63 | 71 | Male | Prostate adenocarcinoma | Anti-PD(L)1 | Overlap Myositis-NMJ disorder (MG) | Not tested | Myocarditis | 3 | 1 | 2 | 2 | 21 | 1 |
| 64 | 58 | Female | Melanoma | Combined anti-PD(L)1 and anti-CTLA4 | Meningitis | Not tested | Uveitis, hepatitis | 3 | 1 | 0 | 0 | 24 | 0 |
| 65 | 59 | Male | Melanoma | Anti-PD(L)1 | Meningitis | Not tested | Uveitis, scleritis | 2 | 1 | 0 | 1 | 18 | 6 |
| 66 | 71 | Male | Renal clear cell carcinoma | Combined anti-PD(L)1 and anti-CTLA4 | Polyradiculoneuropathy | Negative | None | 5 | 2 |  |  | 7 | 2 |
| 67 | 54 | Male | Melanoma | Anti-CTLA4 | Meningoencephalitis | Not tested | Tenosynovitis | 3 | 1 | 1 | 1 | 41 | 1 |
| 68 | 45 | Male | Melanoma | Combined anti-PD(L)1 and anti-CTLA4 | Polyradiculoneuropathy | Negative | Gastritis, colitis | 5 | 6 |  |  | 4 | 6 |
| 69 | 48 | Female | Breast carcinoma | Anti-PD(L)1 | Polyradiculoneuropathy | Negative | Hepatitis | 3 | NA | 3 | 2 | 25 | 3 |
| 70 | 65 | Male | Lung adenocarcinoma | Anti-PD(L)1 | Overlap Myositis-NMJ disorder (MG) | Not tested | None | 3 | 0 | 1 |  | 13 | 1 |
| 71 | 64 | Female | Melanoma | Combined anti-PD(L)1 and anti-CTLA4 | Myelitis | Not tested | Hepatitis | 3 |  |  |  | 3 | 1 |
| 72 | 69 | Male | Prostate adenocarcinoma | Anti-PD(L)1 | Myositis | Not tested | Myocarditis | 6 |  |  |  | 0.5 | 6 |
| 73 | 27 | Male | Melanoma | Anti-PD(L)1 | Cranial neuritis | Not tested | Vitiligo | 2 | 0 | 0 | 0 | 24 | 0 |
| 74 | 51 | Male | Renal clear cell carcinoma | Combined anti-PD(L)1 and anti-CTLA4 | Cranial neuritis | Not tested | None | 1 | 0 | 0 |  | 15 | 0 |
| 75 | 82 | Female | Urothelial bladder carcinoma | Anti-PD(L)1 | NMJ disorder (MG) | Not tested | None | 2 | 1 | 1 |  | 11 | 1 |
| 76 | 76 | Female | Renal clear cell carcinoma | Combined anti-PD(L)1 and anti-CTLA4 | Myositis | Not tested | Myocarditis | 2 | 1 |  |  | 6 | 1 |
| 77 | 74 | Male | Renal clear cell carcinoma | Anti-PD(L)1 | Meningitis | Negative | None | 2 | 2 | 2 |  | 11 | 2 |
| 78 | 66 | Male | Small cell lung cancer | Anti-PD(L)1 | Limbic encephalitis | Hu | None | 5 | 6 |  |  | 4 | 6 |
| 79 | 72 | Male | Renal clear cell carcinoma | Anti-PD(L)1 | Isolated seizures | GAD65 | None | 3 |  |  |  | 1 | 1 |
| 80 | 74 | Male | Melanoma | Anti-PD(L)1 | Myositis | Not tested | Myocarditis | 0 | 0 | 0 | 0 | 17 | 0 |
| 81 | 84 | Male | Squamous cell carcinoma of the skin | Anti-PD(L)1 | Myositis | Not tested | None | 2 | 6 |  |  | 3 | 6 |
| 82 | 34 | Female | Hodgkin lymphoma | Anti-PD(L)1 | Sensory neuronopathy | Not tested | None | 3 | 3 | 3 |  | 15 | 3 |
| 83 | 56 | Male | Squamous cell lung cancer | Anti-PD(L)1 | Myelitis | Unknown antigen | None | 4 | 4 | 4 |  | 14 | 5 |
| 84 | 50 | Male | Renal clear cell carcinoma | Anti-PD(L)1 | Meningoencephalitis | Negative | Skin vasculitis, hypophysitis | 5 | 1 | 2 | 2 | 19 | 2 |
| 85 | 68 | Male | Melanoma | Combined anti-PD(L)1 and anti-CTLA4 | Polyradiculoneuropathy | Not tested | Colitis | 4 | 2 | 2 | 2 | 27 | 2 |
| 86 | 48 | Male | Lung adenocarcinoma | Anti-PD(L)1 | Polyradiculoneuropathy | Not tested | None | 3 | 2 | 2 | 2 | 19 | 2 |
| 87 | 86 | Female | Melanoma | Anti-PD(L)1 | Overlap Myositis-NMJ disorder (MG) | Not tested | None | 4 | 2 | 6 |  | 12 | 6 |
| 88 | 53 | Female | Thymic squamous cell carcinoma | Anti-PD(L)1 | Myositis | Not tested | None | 5 | 2 | 2 |  | 12 | 2 |
| 89 | 31 | Male | Thymic carcinoma | Anti-PD(L)1 | Overlap Myositis-NMJ disorder (MG) | Not tested | Pericarditis, vitiligo | 2 | 1 | 0 | 0 | 32 | 0 |
| 90 | 61 | Male | Renal clear cell carcinoma | Anti-PD(L)1 | Myositis | Not tested | Myocarditis | 6 |  |  |  | 0.5 | 6 |
| 91 | 42 | Female | Melanoma | Combined anti-PD(L)1 and anti-CTLA4 | Meningitis | Negative | None | 3 | 0 | 0 | 0 | 31 | 0 |
| 92 | 70 | Male | Melanoma | Combined anti-PD(L)1 and anti-CTLA4 | Polyradiculoneuropathy | Not tested | Pancytopenia | 4 | 2 | 2 | 6 | 15 | 6 |
| 93 | 42 | Female | Melanoma | Combined anti-PD(L)1 and anti-CTLA4 | Polyradiculoneuropathy | Negative | Colitis, hepatitis | 3 |  |  |  | 1 | 1 |
| 94 | 80 | Female | Lung adenocarcinoma | Anti-PD(L)1 | Myositis | Not tested | Myocarditis | 3 |  |  |  | 3 | 2 |
| 95 | 68 | Male | Melanoma | Anti-PD(L)1 | Myositis | Not tested | None | 4 | 0 | 0 | 0 | 29 | 0 |
| 96 | 49 | Male | Large cell neuroendocrine carcinoma | Anti-PD(L)1 | Myositis | Not tested | None | 3 | 2 | 1 | 6 | 14 | 6 |
| 97 | 68 | Male | Melanoma |  | Myositis | Not tested | None | 3 | 1 | 1 | 1 | 39 | 1 |
| 98 | 66 | Female | Lung adenocarcinoma | Anti-PD(L)1 | Myositis | Not tested | Myocarditis | 4 |  |  |  | 2 | 2 |
| 99 | 59 | Female | Lung adenocarcinoma | Anti-PD(L)1 | Myositis | Not tested | Thyroiditis, myocarditis, pericarditis | 3 | 2 | 2 |  | 13 | 2 |
| 100 | 48 | Female | Colon adenocarcinoma | Combined anti-PD(L)1 and anti-CTLA4 | Cranial neuritis | Not tested | Uveitis | 4 | 1 | 1 | 1 | 29 | 1 |
| 101 | 68 | Male | Melanoma | Anti-PD(L)1 | Myositis | Not tested | None | 2 |  |  |  | 2 | 1 |
| 102 | 69 | Male | Squamous cell lung cancer | Anti-PD(L)1 | Myositis | Not tested | Myocarditis | 3 | 2 | 6 |  | 13 | 6 |
| 103 | 58 | Male | Melanoma | Anti-PD(L)1 | Myositis | Not tested | Hepatitis | 3 | 1 | 0 | 0 | 17 | 0 |
| 104 | 67 | Female | Lung adenocarcinoma | Anti-PD(L)1 | Myositis | Not tested | Myocarditis | 3 | 1 | 1 |  | 14 | 1 |
| 105 | 53 | Female | Renal clear cell carcinoma | Combined anti-PD(L)1 and anti-CTLA4 | Myositis | Not tested | Myocarditis | 2 | 0 | 1 |  | 14 | 1 |
| 106 | 64 | Female | Colon adenocarcinoma | Combined anti-PD(L)1 and anti-CTLA4 | Polyradiculoneuropathy | Not tested | None | 3 | 3 | 0 |  | 12 | 0 |
| 107 | 58 | Male | Melanoma | Combined anti-PD(L)1 and anti-CTLA4 | Myelitis | Unknown antigen | None | 4 | 4 | 3 |  | 14 | 3 |
| 108 | 43 | Male | Lung adenocarcinoma | Combined anti-PD(L)1 and anti-CTLA4 | Myositis | Not tested | Pericarditis | 2 | 0 | 0 |  | 14 | 1 |
| 109 | 80 | Male | Melanoma | Anti-PD(L)1 | Myositis | Not tested | Myocarditis | 3 | 1 | 1 |  | 10 | 1 |
| 110 | 73 | Male | Squamous cell carcinoma of the skin | Anti-PD(L)1 | Myositis | Not tested | Myocarditis | 2 | 0 |  |  | 7 | 0 |
| 111 | 25 | Male | Thymoma | Anti-PD(L)1 | Myositis | Not tested | Myocarditis | 2 | 0 |  |  | 8 | 0 |
| 112 | 72 | Male | Renal clear cell carcinoma | Combined anti-PD(L)1 and anti-CTLA4 | Polyradiculoneuropathy | Not tested | Thyroiditis, hypophysitis, nephritis | 2 | 1 |  |  | 6 | 1 |
| 113 | 66 | Female | Lung adenocarcinoma | Anti-PD(L)1 | Sensory neuronopathy | Not tested | None | 4 | 3 | 3 | NA | 35 | 3 |
| 114 | 63 | Male | Oropharyngeal squamous cell carcinoma | Anti-PD(L)1 | Myositis | Not tested | Myocarditis | 4 |  |  |  | 2 | 3 |
| 115 | 84 | Male | Lung adenocarcinoma | Anti-PD(L)1 | Myositis | Not tested | Myocarditis | 4 | 6 |  |  | 3 | 6 |
| 116 | 67 | Male | Lung adenocarcinoma | Anti-PD(L)1 | Overlap Myositis-NMJ disorder (MG) | Not tested | Myocarditis | 3 | 2 | 6 |  | 11 | 6 |
| 117 | 49 | Female | Acute myeloblastic leukemia | Anti-PD(L)1 | Myositis | Not tested | None | 3 | 1 | 1 | 1 | 23 | 1 |
| 118 | 76 | Male | Renal clear cell carcinoma | Anti-PD(L)1 | Myositis | Not tested | Myocarditis | 4 | 6 |  |  | 1 | 6 |
| 119 | 59 | Female | Melanoma | Combined anti-PD(L)1 and anti-CTLA4 | Polyradiculoneuropathy | Not tested | Cutaneous sarcoidosis | 3 | 1 |  |  | 5 | 1 |
| 120 | 82 | Female | Squamous cell carcinoma of the skin | Anti-PD(L)1 | Myositis | Not tested | Myocarditis | 5 | 4 |  |  | 7 | 4 |
| 121 | 79 | Male | Lung adenocarcinoma | Anti-PD(L)1 | Myositis | Not tested | Myocarditis | 5 | 4 | 6 |  | 10 | 6 |
| 122 | 54 | Male | Melanoma | Combined anti-PD(L)1 and anti-CTLA4 | Myositis | Not tested | Myocarditis | 2 |  |  |  | 3 | 3 |
| 123 | 81 | Female | Urothelial bladder carcinoma | Anti-PD(L)1 | Myositis | Not tested | None | NA |  |  |  | 1 | NA |
| 124 | 84 | Female | Colon adenocarcinoma | Anti-PD(L)1 | Myositis | Not tested | Myocarditis | 4 |  |  |  | 2 | 3 |
| 125 | 70 | Female | Lung squamous cell carcinoma | Anti-PD(L)1 | Myositis | Not tested | Thyroiditis | NA | NA | NA | 4 | 21 | 4 |
| 126 | 62 | Female | Squamous cell lung cancer | Combined anti-PD(L)1 and anti-CTLA4 | Myositis | Not tested | Myocarditis | 3 | 1 | 1 |  | 13 | 3 |
| 127 | 63 | Female | Melanoma | Anti-PD(L)1 | Small fiber neuropathy | Unknown antigen | None | 4 | 3 | 3 | 3 | 21 | 3 |
| 128 | 80 | Female | Melanoma | Anti-PD(L)1 | Myositis | Not tested | Myocarditis | 6 |  |  |  | 0.5 | 6 |
| 129 | 65 | Male | Lung adenocarcinoma | Anti-PD(L)1 | Polyradiculoneuropathy | Not tested | Colitis | 6 |  |  |  | 1 | 6 |
| 130 | 42 | Female | Melanoma | Combined anti-PD(L)1 and anti-CTLA4 | Meningitis | Not tested | None | 3 | 1 | 0 | 0 | 31 | 0 |
| 131 | 65 | Female | Melanoma | Combined anti-PD(L)1 and anti-CTLA4 | Meningoencephalitis | Not tested | None | 5 | 1 | NA | 6 | 14 | 6 |
| 132 | 20 | Female | Melanoma | Combined anti-PD(L)1 and anti-CTLA4 | Meningitis | Not tested | None | 2 | 0 | 0 | 0 | 32 | 0 |
| 133 | 50 | Female | Melanoma | Combined anti-PD(L)1 and anti-CTLA4 | Small fiber neuropathy | Negative | None | 4 | 3 | 2 | 3 | 16 | 3 |
| 134 | 72 | Female | Small cell lung cancer | Anti-PD(L)1 | Sensory neuronopathy | Negative | None | 4 | 4 |  |  | 5 | 4 |
| 135 | 84 | Female | Hepatocellular carcinoma | Anti-PD(L)1 | Myositis | Not tested | Myocarditis | 5 | 6 |  |  | 3 | 6 |
| 136 | 80 | Male | Urothelial bladder carcinoma | Anti-PD(L)1 | Myositis | Not tested | Myocarditis | 6 |  |  |  | 1 | 6 |
| 137 | 70 | Female | Small cell lung cancer | Anti-PD(L)1 | Cranial neuritis | Unknown antigen | None | 4 | 3 |  |  | 5 | 3 |
| 138 | 72 | Male | Melanoma | Anti-PD(L)1 | Myositis | Not tested | None | 1 | 0 | 0 |  | 13 | 0 |
| 139 | 50 | Male | Melanoma | Combined anti-PD(L)1 and anti-CTLA4 | Myositis | Not tested | Myocarditis | 2 |  |  |  | 3 | 0 |
| 140 | 87 | Male | Melanoma | Anti-PD(L)1 | Myositis | Not tested | Dermatitis | 1 | NA | 0 | 0 | 20 | 0 |
| 141 | 86 | Male | Merkel carcinoma | Anti-PD(L)1 | Myositis | Not tested | None | 1 | 0 | 1 | 0 | 45 | 1 |
| 142 | 61 | Male | Melanoma | Combined anti-PD(L)1 and anti-CTLA4 | Myositis | Not tested | None | 3 | NA | 6 |  | 10 | 6 |
| 143 | 72 | Male | Renal clear cell carcinoma | Combined anti-PD(L)1 and anti-CTLA4 | Meningoencephalitis | Not tested | None | 6 |  |  |  | 0.5 | 6 |
| 144 | 68 | Male | Small cell lung cancer | Anti-PD(L)1 | Brainstem encephalitis, polyradiculoneuropathy | Hu | None | 6 |  |  |  | 1 | 6 |
| 145 | 65 | Female | Melanoma | Combined anti-PD(L)1 and anti-CTLA4 | Polyradiculoneuropathy | Unknown antigen | None | 4 |  |  |  | 0.5 | 4 |
| 146 | 79 | Male | Melanoma | Combined anti-PD(L)1 and anti-CTLA4 | Cerebellitis | Negative | Vitiligo, thyroiditis | 3 |  |  |  | 0.5 | 2 |
| 147 | 44 | Female | Small cell lung cancer | Anti-PD(L)1 | Limbic encephalitis | Hu | None | 4 |  |  |  | 1 | 4 |

Abbreviations: CTLA4 = Cytotoxic T-Lymphocyte Antigen 4; ICI = immune checkpoint inhibitor; mRS = modified Rankin Score; n-irAEs = neurological immune-related adverse events; PD1 = Programmed Cell Death Protein 1; Programmed death-ligand 1 = PDL1.

**Supplementary Table 2. Cancer types.**

| **Cancer types** | **N (%)** |
| --- | --- |
| Lung  NSCLC  SCLC | 53 (36.1)  42 (28.6)  11 (7.5) |
| Melanoma | 45 (30.5) |
| Urinary tract  Clear cell renal cell carcinoma  Urothelial bladder carcinoma | 23 (15.5)  18 (12.2)  5 (3.4) |
| Others  Thymoma/thymic carcinoma*  Hodgkin lymphoma  Colon carcinoma  Carcinoma of the skin  Prostate carcinoma  Merkel carcinoma  Oropharyngeal carcinoma  Breast carcinoma  Mesothelioma  Chordoma  Ovarian carcinoma  Retroperitoneal leiomyosarcoma  Acute myeloblastic leukemia*  Spleen liposarcoma*  Hepatocellular carcinoma | 26 (17.7)  4 (2.7)  3 (2.0)  3 (2.0)  3 (2.0)  2 (1.4)  2 (1.4)  1 (0.7)  1 (0.7)  1 (0.7)  1 (0.7)  1 (0.7)  1 (0.7)  1 (0.7)  1 (0.7)  1 (0.7) |

Abbreviations: NSCLC = non-small-cell lung cancer; SCLC = small-cell lung cancer.

*ICI currently not approved and administered under a “Temporary Authorization for Use”, a French compassionate program which allows the use of drugs before their marketing authorization.

**Supplementary Table 3. Immune checkpoint inhibitors.**

|  | Lung  n=53 | Melanoma  n=45 | Urinary tract  n=23 | Others  n=26 |
| --- | --- | --- | --- | --- |
| Immune Checkpoint Inhibitors  PD1 Inhibitors  Nivolumab  Pembrolizumab  Cemiplimab  PDL1 Inhibitors  Atezolizumab  Avelumab  Durvalumab  CTLA4 Inhibitors  Ipilimumab  Combined PD(L)1 and CTLA4 Inhibitors  Nivolumab and ipilimumab  Pembrolizumab and ipilimumab  Durvalumab and tremelimumab  Unknown* | 16 (30.2)  23 (43.4)  0  7 (13.2)  0  5 (9.4)  0  2 (3.8)  0  0  0 | 6 (13.3)  9 (20.0)  0  0  0  0  5 (11.1)  21 (46.7)  3 (6.7)  0  1 (2.2) | 8 (34.8)  6 (26.1)  0  0  0  0  0  8 (34.8)  0  1 (4.4)  0 | 7 (26.9)  7 (26.2)  3 (11.5)  2 (7.7)  3 (11.5)  1 (3.8)  0  3 (11.5)  0  0  0 |

*Information blinded: nivolumab alone or nivolumab and ipilimumab.

Abbreviations: CTLA4 = cytotoxic T-lymphocyte antigen-4; PD1 = programmed death 1; PDL1 = programmed death ligand 1.

**Supplementary Table 4. Characteristics of patients who relapsed.**

| **Age (years)** | **Sex** | **Cancer** | **ICI** | **Phenotype** | **Antineural antibodies** | **ICI rechallenge** | **Time of relapse since the first event (months)** |
| --- | --- | --- | --- | --- | --- | --- | --- |
| 79 | Male | Lung adenocarcinoma | Pembrolizumab | Limbic encephalitis | Ma2 | No | 9 |
| 72 | Male | Lung adenocarcinoma | Nivolumab | Opsoclonus myoclonus | Unknown antigen | No | 18 |
| 70 | Male | Small cell lung cancer | Atezolizumab | Cerebellitis | Hu | No | 7 |
| 57 | Male | Lung adenocarcinoma | Nivolumab | Myelitis | Negative | No | 5 |
| 72 | Male | Renal clear cell carcinoma | Nivolumab and ipilimumab | Meningoencephalitis and polyradiculoneuropathy | Unknown antigen | No | 4 |
| 70 | Male | Mesothelioma | Nivolumab and ipilimumab | Limbic encephalitis | Ma2 | No | 6 |
| 45 | Male | Melanoma | Nivolumab and ipilimumab | Polyradiculoneuropathy | Negative | No | 4 |
| 56 | Male | Squamous cell lung cancer | Nivolumab | Myelitis | Unknown antigen | No | 5 |
| 49 | Male | Large cell neuroendocrine carcinoma | Nivolumab | Myositis | Not tested | No | 2 |
| 64 | Female | Colon adenocarcinoma | Nivolumab and ipilimumab | Polyradiculoneuropathy | Not tested | Nivolumab | 6 |
| 65 | Female | Melanoma | Nivolumab and ipilimumab | Meningoencephalitis | Not tested | Pembrolizumab | 8 |
| 50 | Female | Melanoma | Nivolumab and ipilimumab | Small-fiber neuropathy | Negative | No | 14 |

Abbreviations: ICI = immune checkpoint inhibitor.

**SUPPLEMENTARY FIGURES**

**Supplementary Figure 1. UpSet plot indicating combinations of phenotypes in 21 patients with more than one identifiable neurological phenotype.**

**Supplementary Figure 2. Median time from first immune checkpoint inhibitor (ICI) dose to neurological immune-related adverse event (n-irAE) onset according to the clinical phenotype (n=145).**

**Supplementary Figure 3. Neuronal/glial antibody results according to the clinical phenotype.**

Results are expressed as absolute values. Neuronal/glial antibodies were tested in 73/147 (49.7%) patients (45/73, 61.6% in serum; 66/73, 90.4% in CSF), mostly in patients with central nervous system involvement and peripheral neuropathies, and only in 2 cases with myositis/neuromuscular junction disorders. Paraneoplastic-related antibodies included Ma2 (n=9), Hu (n=7), SOX1 (n=3), Yo (n=3), CV2/CRMP5 (n=1), Ri (n=1) and were mostly (19/24, 79.2%) found in patients with paraneoplastic-like syndromes (limbic encephalitis, rapidly progressive cerebellar ataxia, sensory neuronopathy, and Lambert-Eaton). Conversely, GFAP antibodies were found in 6 patients with meningoencephalitis, two of them also had myelitis. Antibodies found in less than 3 patients are not represented (CASPR2, n=2; AGO2, n=1; TRIM9, n=1; GAD65, n=1).

**Supplementary Figure 4.** **Common Terminology Criteria for Adverse Events (CTCAE) at onset and last visit (overall cohort).**

**
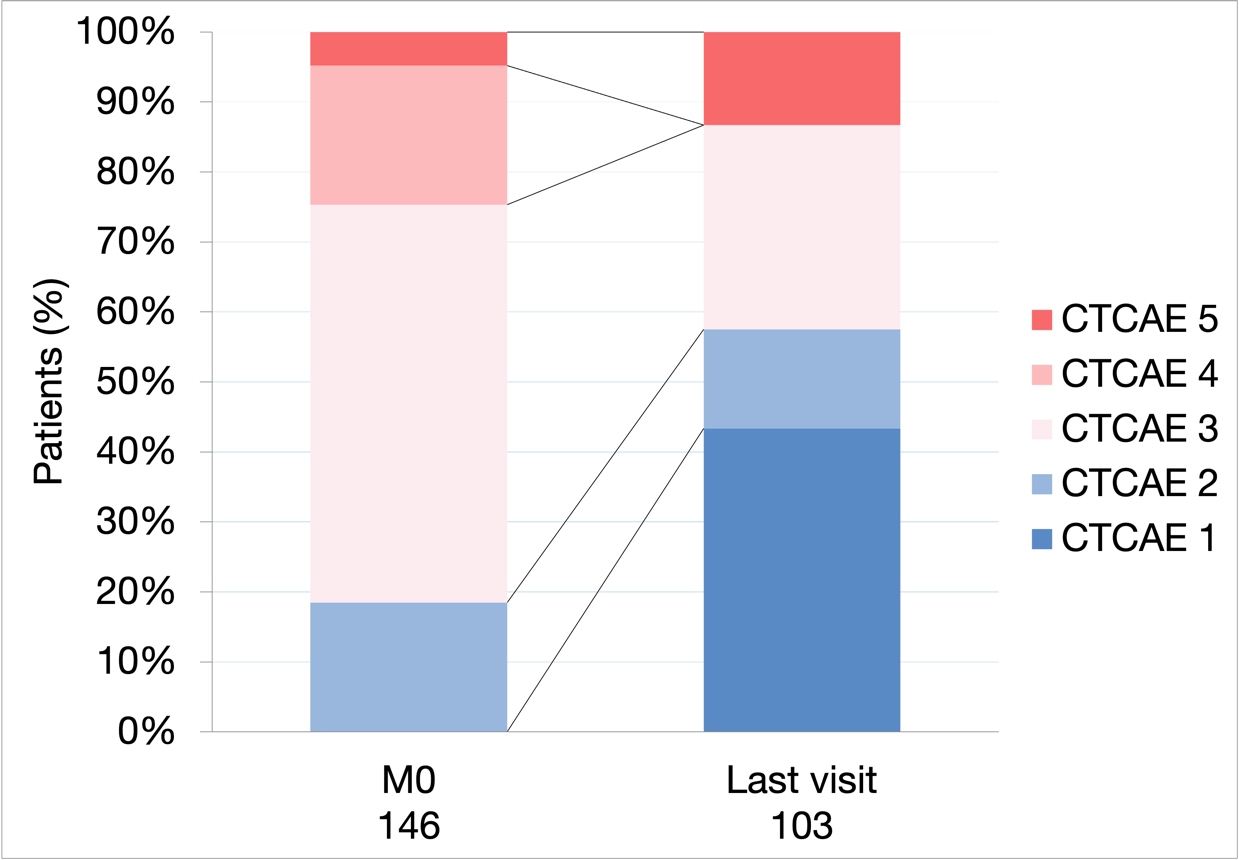
**

Disability at onset (M0) and last visit (median follow-up 12 months, range 0.5-50). At onset, 27/146 patients (18.5%) had CTCAE grade 2, 83/146 (56.8%) CTCAE grade 3, 29/146 (19.9%) CTCAE grade 4, and 7/146 (4.8%) CTCAE grade 5. At last visit, 49/113 patients (43.4%) had CTCAE grade 1, 16/113 (14.2%) CTCAE grade 2, 33/113 (29.2%) CTCAE grade 3, and 15/113 (13.3%) CTCAE grade 5. Patient who died from causes other than n-irAEs (n=27) or unknown causes (n=6) were not included in this analysis.

**Supplementary Figure 5. Common Terminology Criteria for Adverse Events (CTCAE) at last visit according to the clinical presentation.**

**
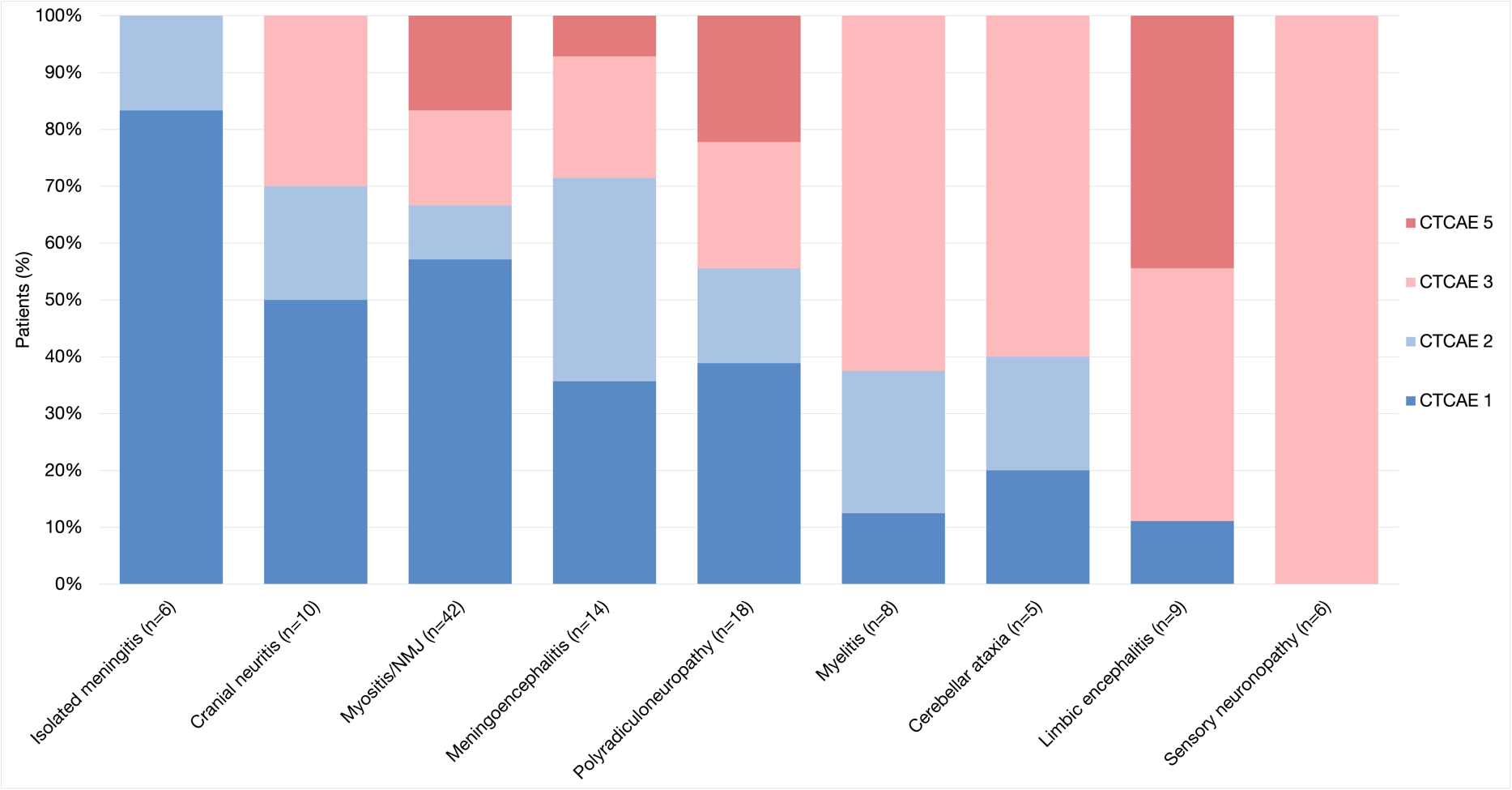
**

Outcome at last visit (median (range) follow-up duration: 12, (0.5-50) months) according to the initial clinical presentation. Less frequent phenotypes (small-fiber neuropathy, n=2, length-dependent polyneuropathy, n=1, chronic intestinal pseudo-obstruction, n=1, neuromyotonia, n=1) are not represented. Patient who died from causes other than n-irAEs (n=27) or unknown causes (n=6) were not included in this analysis.

**Supplementary Figure 6. Markov model for long-term outcomes: results of the univariate analysis**

**
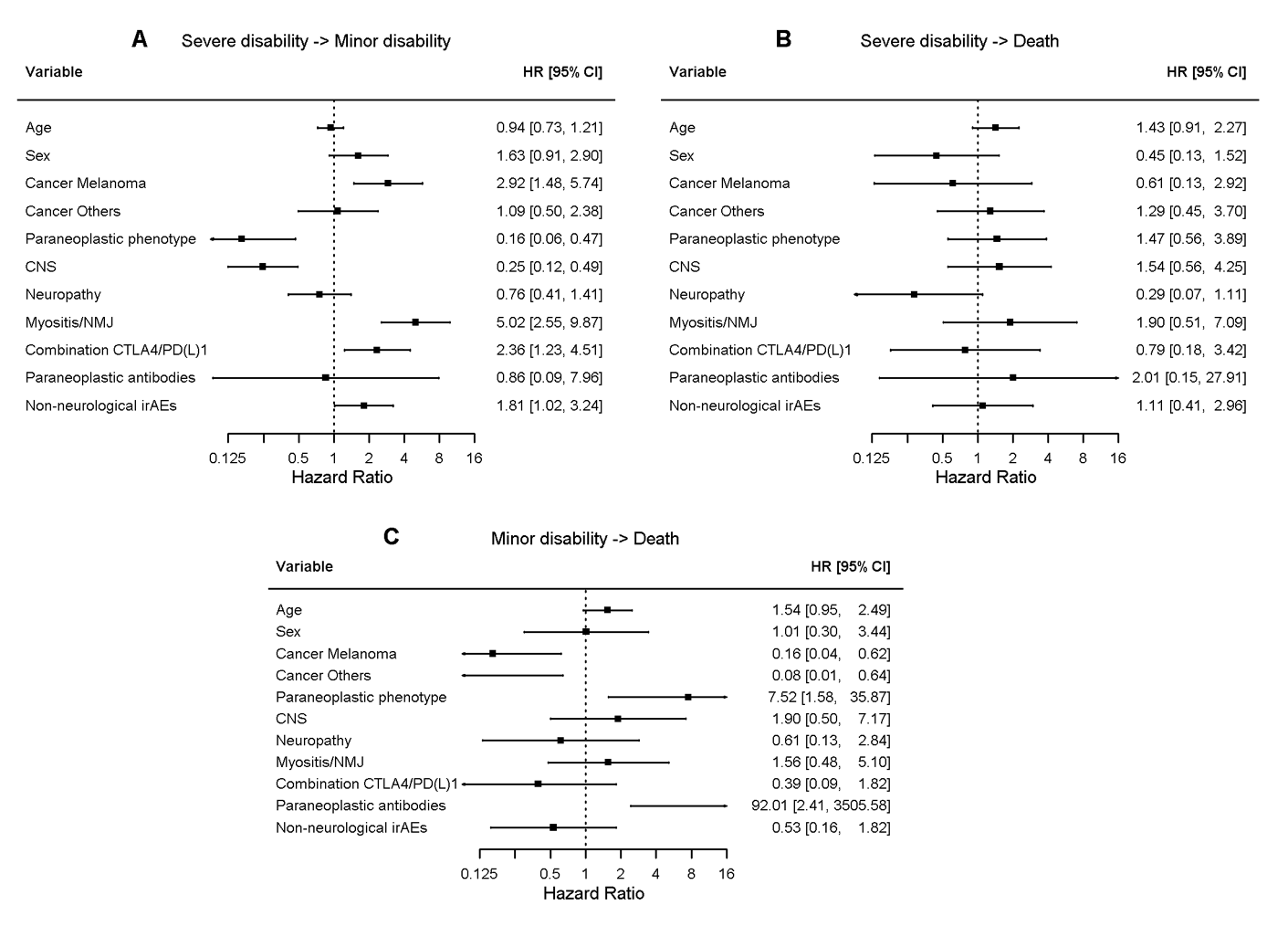
**

Forest plots showing the hazard ratios and confidence intervals for the probabilities of transition (114 patients included in this analysis) from, respectively: A, severe disability (mRS 3-5) to minor disability (mRS≤2); B, severe disability to death; and C, minor disability to death. Univariate analyses identified paraneoplastic-like syndromes and central nervous system involvement as negatively associated with transition from severe to minor disability, while myositis/NMJ disorder, melanoma (compared with lung cancer), combined PD(L)1 / CTLA4 therapy, and co-occurrence of non-neurological irAEs, increased the transition rate towards minor disability. Paraneoplastic-like syndromes and paraneoplastic-related antibodies were associated with a higher transition rate from minor disability to death, while melanoma and cancers other than lung and melanoma decreased this rate (compared to lung cancer).

Abbreviations: CI = confidence interval; HR = hazard ration; mRS = modified Rankin scale; NMJ = neuromuscular junction.
